# HMGN1 and HMGN2 are recruited to acetylated and histone variant H2A.Z-containing nucleosomes to regulate chromatin state and transcription

**DOI:** 10.1016/j.jbc.2025.110997

**Published:** 2025-11-29

**Authors:** Riya Gohil, Zhihan Gao, Rebecca A. Lewis, Nathaniel T. Burkholder, Brian D. Strahl, Jill M. Dowen

**Affiliations:** 1 Curriculum in Genetics and Molecular Biology, University of North Carolina at Chapel Hill, Chapel Hill, North Carolina, USA; 2 Integrative Program for Biological and Genome Sciences, University of North Carolina at Chapel Hill, Chapel Hill, North Carolina, USA; 3 Department of Biology, University of North Carolina at Chapel Hill, Chapel Hill, North Carolina, USA; 4 Department of Biochemistry & Biophysics, University of North Carolina at Chapel Hill, Chapel Hill, North Carolina, USA; 5 Lineberger Comprehensive Cancer Center, University of North Carolina at Chapel Hill, Chapel Hill, North Carolina, USA

**Keywords:** histone acetylation, chromatin, gene expression, nucleosome, epigenetics, E1A binding protein p300, genome structure, high mobility group proteins

## Abstract

The High Mobility Group Nucleosome-binding (HMGN) proteins are small, abundant nuclear proteins that directly bind nucleosomes and form a major component of chromatin. HMGN proteins localize to enhancers and actively transcribed genes across the genome; however, their roles in regulating chromatin structure and transcription remain poorly understood. Although it is well established that HMGN proteins bind to the H2A–H2B acidic patch on nucleosomes, other potential nucleosome targeting mechanisms, including histone post-translational modifications and histone variants, remain unclear. To investigate the nucleosomal binding preferences and function of HMGN proteins, we engineered mouse embryonic stem cells (mESCs) lacking HMGN1 and/or HMGN2 (*Hmgn1*^*−/−*^ mESCs, *Hmgn2*^*−/−*^ mESCs, and *Hmgn1*^*−/−*^*Hmgn2*^*−/−*^ mESCs) and profiled gene expression and localization of architectural proteins. In the absence of these HMGN proteins, ∼1000 genes were differentially expressed, including cell identity genes, with most genes being downregulated. Nucleosome binding assays revealed preferential binding of HMGN1 and HMGN2 proteins to nucleosomes with acetylated H3 tail residues and nucleosomes containing the histone variant H2A.Z. In addition, *in vitro* acetylation assays demonstrated that binding of HMGN1 and HMGN2 to nucleosomes reduces p300-mediated acetylation of H3K18, H3K23, and H3K27 residues. An epiproteomic mass spectrometry analysis of histone tail modifications revealed that *Hmgn1*^*−/−*^*Hmgn2*^*−/−*^ mESCs have increased steady-state levels of H3K27me2 and H3K27me3, but not H3 tail acetylation, relative to WT cells. Together, these findings show that HMGN proteins function as both sensors and modulators of the histone post-translational modification landscape inside cells, playing a critical role in the dynamic balance between active and repressive chromatin states.

A central question in the field of chromatin biology is how distinct chromatin states are molecularly defined and dynamically regulated. Major contributors to chromatin dynamics and the establishment of chromatin states include histone post-translational modifications (PTMs), histone variants, and other molecular machines, such as chromatin remodelers that space and regulate nucleosome structure. Additional proteins further contribute to this regulation through the formation of DNA loops and topologically associating domains, both of which are correlated with transcription of genes (1, 2, 3, 4, 5). The mechanisms that regulate chromatin and underlie proper gene expression are active areas of investigation. Transcription is regulated by dynamic changes in chromatin and recruitment of chromatin-associating proteins to specific genomic sites (6). Despite extensive study of chromatin dynamics, there are many unresolved questions regarding how chromatin-associating proteins localize to specific sites to regulate gene expression in a dynamic, context-dependent manner. One such class of chromatin-associating proteins that have gained increasing attention for their roles in chromatin regulation are the High Mobility Group Nucleosome-binding (HMGN) proteins.

HMGN proteins play key roles in chromatin structure, transcriptional regulation, and disease pathogenesis, including numerous cancers (7, 8, 9, 10, 11, 12, 13, 14). HMGN proteins are abundant nucleosome-binding proteins, with an estimated ratio of one HMGN protein for every 100 nucleosomes in chromatin (15). HMGN proteins contain a nucleosome-binding domain (NBD) that interacts with the residues of the acidic patch on the nucleosome “disk face” formed by H2A and H2B (9). While many proteins have been found to be direct acidic patch–dependent nucleosome-binding proteins, HMGN2 was recently shown to be one of the strongest interactors with the nucleosome acidic patch (16). Methyl-based NMR analysis identified a conserved arginine-rich region within the NBD of HMGN proteins that binds to the H2A–H2B acidic patch and a lysine-rich region within the NBD of HMGN proteins that makes contact with DNA near the DNA entry/exit point of the nucleosome (17). This study also suggested that the C-terminal tail of HMGN proteins resides proximal to the H3 tail, providing an opportunity for HMGN protein recruitment to nucleosomes to be sensitive to the state of histone tails (17, 18).

Importantly, HMGN proteins are not uniformly distributed across the genome but are specifically enriched at transcriptionally active *cis*-regulatory elements, such as enhancers and promoters (19, 20, 21, 22, 23, 24). Previous studies have shown that HMGN proteins localize to H3K27ac-containing nucleosomes in the genome; however, HMGN proteins neither possess conventional chromatin reader domains nor have DNA-binding domains (8, 13, 17, 20). How HMGN proteins are recruited to active chromatin and their functions at these sites remain unclear. HMGN proteins are thought to influence gene regulation through their ability to modulate chromatin structure (19, 25, 26, 27, 28). HMGN1 and HMGN2 proteins promote chromatin accessibility at enhancers and promoters, thus allowing for transcription factor binding at these sites (19, 29). Molecular understanding of how HMGN proteins regulate chromatin dynamics and facilitate the binding of chromatin regulatory complexes at enhancers and promoters remains unclear.

To investigate the role of HMGN proteins in chromatin regulation, we investigated the localization of HMGN1 and HMGN2 proteins relative to other key regulators of transcription and enhancer–promoter chromatin architecture. HMGN1 and HMGN2 occupy 86% of actively transcribed genes in the mouse embryonic stem cell (mESC) genome, which contain H3K27ac, H3K4me3, H2A.Z, and the architectural proteins cohesin and CTCF. We engineered mESCs lacking HMGN1 and/or HMGN2 (*Hmgn1*^*−/−*^ mESCs, *Hmgn2*^*−/−*^ mESCs, and *Hmgn1*^*−/−*^*Hmgn2*^*−/−*^ mESCs) and profiled gene expression to investigate the function of HMGN proteins at cell identity genes and found that loss of HMGN1 and HMGN2 leads to the downregulation of many cell identity genes. Furthermore, the binding of HMGN proteins to nucleosomes with various histone PTMs or histone variants was analyzed using a high-throughput and sensitive multiplex platform. The results show that HMGN1 and HMGN2 preferentially bind to nucleosomes containing the H2A.Z variant, as well as nucleosomes with acetylated histone H3 tail residues, shedding light on the specific molecular features that regulate the localization of HMGN proteins to active chromatin. Intriguingly, we find that nucleosome-bound HMGN1 and HMGN2 limit p300-mediated acetylation of the histone H3 tail residues K18, K23, and K27, which suggests HMGN proteins may not only respond to the acetylated histone landscape but also act to regulate the extent to which nucleosomes are acetylated. Loss of HMGN proteins *in vivo* leads to a global increase in histone methylation at H3K27 and downregulation of hundreds of cell identity genes. These findings open new avenues of exploration regarding the molecular function(s) of HMGN proteins and their contributions to gene expression and chromatin state in health and disease, particularly in cancer.

## Results

### HMGN proteins localize to active regions of the genome

**Figure 1:**
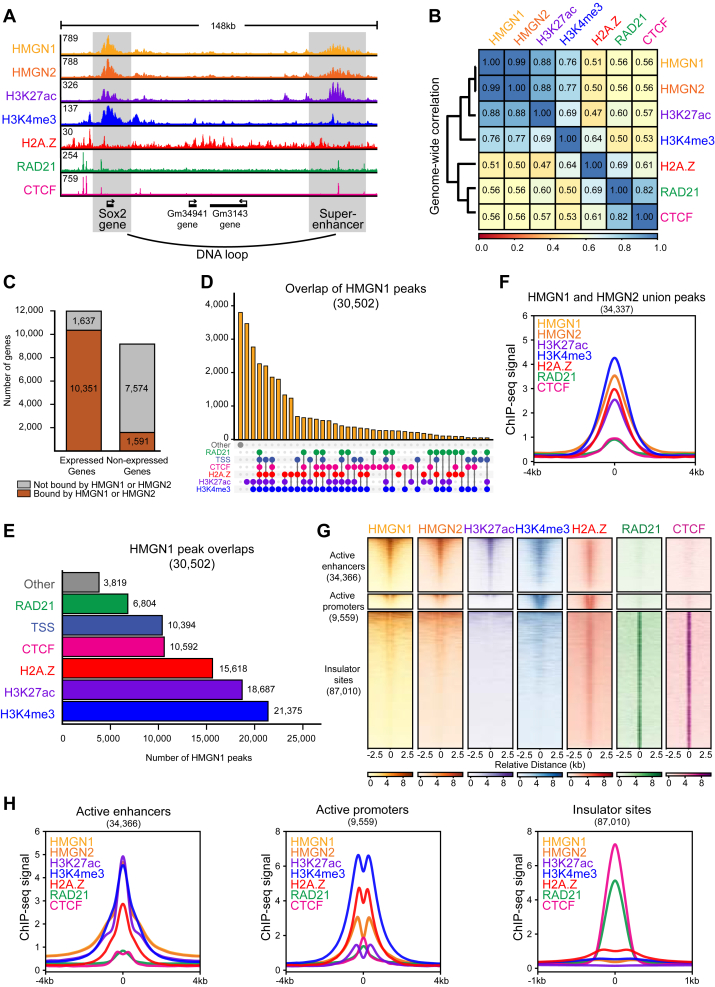
**HMGN proteins localize to transcriptionally active regions of the genome**. *A*, genome browser tracks of HMGN1, HMGN2, H3K27ac, H3K4me3, H2A.Z, RAD21, and CTCF ChIP-Seq signal at the promoter of Sox2 and the super-enhancer domain downstream of Sox2 in WT mESCs. *B*, Pearson’s correlation hierarchical clustering heatmap of genome-wide signal of HMGN1, HMGN2, H3K27ac, H3K4me3, H2A.Z, RAD21, and CTCF ChIP-Seq datasets in WT mESCs. *C*, bar graph of the number of expressed genes and non-expressed genes in the mouse embryonic stem cell (mESC) genome bound and not bound by HMGN1 and HMGN2. Active genes are defined as genes with a RPKM value ≥22 as defined by the EMBL Expression Atlas. *D*, UpSet plot of HMGN1 ChIP-Seq peaks in WT mESCs displaying intersection of sets of peaks at H3K27ac, H3K4me3, transcription start sites (TSSs), H2A.Z, RAD21, CTCF, and other sites. *E*, bar graph of the number of HMGN1 peaks that overlap with H3K4me3, H3K27ac, CTCF, H2A.Z, TSSs, RAD21, and other peaks in WT mESCs. *F*, average signal plot of HMGN1, HMGN2, H3K27ac, H3K4me3, H2A.Z, RAD21, and CTCF ChIP-Seq signal at a union list of all HMGN1 and HMGN2 peaks (Z-score normalized). *G*, clustered heatmaps of HMGN1, HMGN2, H3K27ac, H3K4me3, H2A.Z, RAD21, and CTCF ChIP-Seq signal at active enhancers, active promoters, and insulator sites, ordered by HMGN2 signal (Z-score normalized). *H*, average signal plots of HMGN1, HMGN2, H3K27ac, H3K4me3, H2A.Z, RAD21, and CTCF ChIP-Seq signal in WT mESCs at active enhancers, active promoters, and insulator sites (Z-score normalized). ChIP-Seq, chromatin immunoprecipitation followed by sequencing; HMGN, High Mobility Nucleosome-binding protein; mESC, mouse embryonic stem cell; RPKM, reads per kilobase of transcript per million mapped reads.

To assess the genome-wide binding pattern of HMGN proteins, we analyzed publicly available chromatin immunoprecipitation followed by high-throughput sequencing (ChIP-Seq) data for HMGN1 and HMGN2, the most highly expressed HMGN proteins in mESCs (Fig. S1, *A*–*C*) (19). The genome-wide binding profiles of HMGN1 and HMGN2 were found to overlap with H3K27ac, H3K4me3, H2A.Z, RAD21 (a core subunit of the cohesin complex), and CTCF (Fig. 1, *A* and *B*, Table S1). HMGN1 and HMGN2 displayed highly similar binding patterns across the genome (Pearson’s correlation value *r* = 0.99) and had a similar number of peaks identified, with 30,502 HMGN1 peaks and 29,593 HMGN2 peaks, respectively (Fig. 1*B*, Table S2). HMGN1 and HMGN2 localized to *cis*-regulatory sites important for gene transcription and chromatin organization that overlap with the histone PTMs H3K27ac and H3K4me3, H2A.Z, and the architectural proteins, RAD21 and CTCF (Fig. 1*A*). Interestingly, HMGN1 and HMGN2 ChIP-Seq signal occurs in broad peaks similar to those of histone PTMs and distinct from the narrow peaks of many DNA-binding factors (Fig. 1*A*). HMGN1 and HMGN2 genome-wide signal was positively correlated with the histone PTMs H3K27ac and H3K4me3, which occur at active enhancers and promoters (Pearson’s correlation coefficient *r* = 0.76–0.88). HMGN1 and HMGN2 genome-wide signal was moderately correlated with H2A.Z (Pearson’s correlation coefficient *r* = 0.50 and 0.51), which is a histone H2A variant found at promoters, and the architectural proteins RAD21 and CTCF (Pearson’s correlation coefficient *r* = 0.56) (Fig. 1*B*). Notably, over 86% of expressed genes in the mESC genome are bound by HMGN1 or HMGN2, whereas only 17% of nonexpressed genes are bound by HMGN1 or HMGN2 (Fig. 1*C*). Furthermore, a large number of HMGN1 and HMGN2 peaks overlap H3K27ac, H3K4me3, transcription start sites (TSSs), H2A.Z, and, to a lesser extent, RAD21 and CTCF, architectural proteins that mediate DNA loops (Fig. 1, *D*–*F*, Fig. S1, *D*–*F*). A *cis*-regulatory centric analysis shows that active enhancers and promoters have strong HMGN1, HMGN2, H3K27ac, H3K4me3, and H2A.Z signal, whereas insulator sites that do not overlap enhancers and promoters do not display H3K27ac, H3K4me3, HMGN1, or HMGN2 signal (Fig. 1, *G* and *H*, Fig. S1*F*). Together, these results demonstrate that HMGN1 and HMGN2 localize to enhancers and promoters of actively expressed genes.

### Loss of HMGN1 or HMGN2 causes downregulation of gene expression

**Figure 2:**
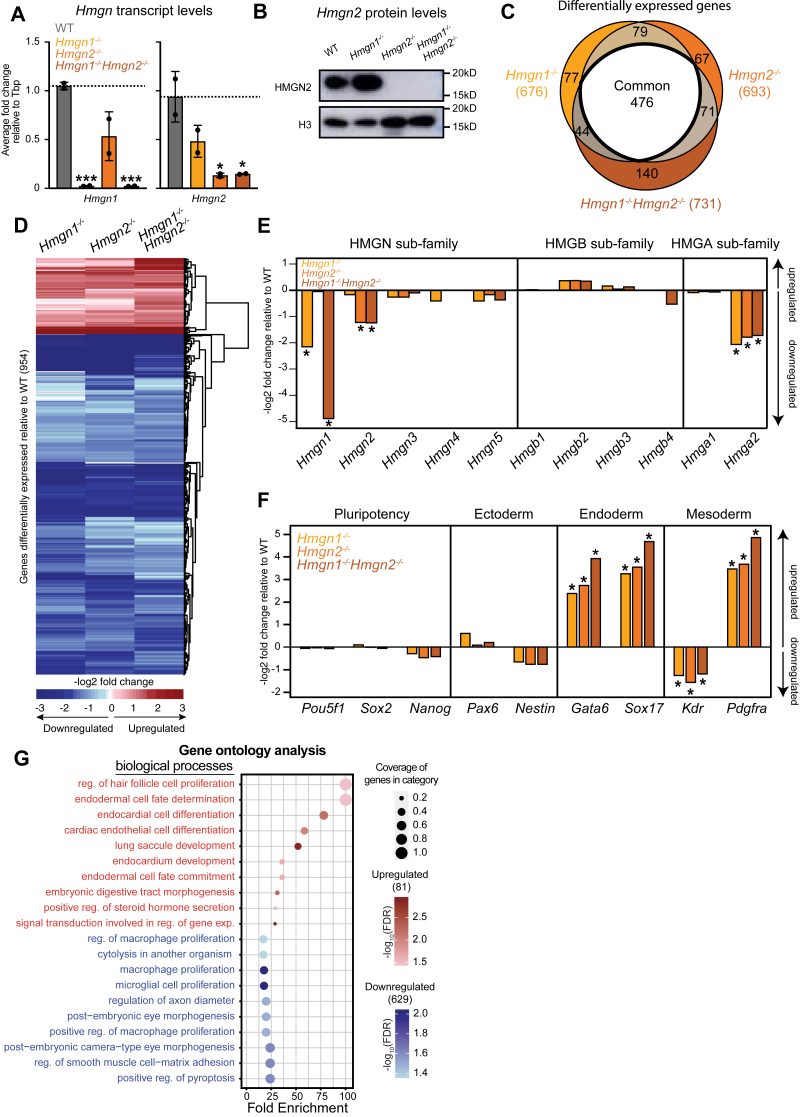
**HMGN1 and HMGN2 are required for maintenance of cell identity gene expression programs**. *A*, bar graphs of average fold change (FC) relative to Tbp in transcript levels of *Hmgn1* and *Hmgn2* in WT mESCs, *Hmgn1*^*−/−*^ mESCs, *Hmgn2*^*−/−*^ mESCs, and *Hmgn1*^*−/−*^*Hmgn2*^*−/−*^ mESCs. Error bars represent the standard deviation calculated from two biological replicates, each consisting of three technical replicates, with two outliers removed from the dataset. A *t* test was used to assess statistical significance, with one *asterisk* (∗) denoting a *p* value less than 0.05, ∗∗ indicating a *p* value less than 0.01, and ∗∗∗ representing a *p* value less than 0.001. *B*, Western blot analysis of nuclear lysates of HMGN2 protein levels in WT mESCs, *Hmgn1*^*−/−*^ mESCs, *Hmgn2*^*−/−*^ mESCs, and *Hmgn1*^*−/−*^*Hmgn2*^*−/−*^ mESCs relative to H3 loading control. *C*, overlap of differentially expressed genes (DEGs) in *Hmgn1*^*−/−*^ mESCs, *Hmgn2*^*−/−*^ mESCs, and *Hmgn1*^*−/−*^*Hmgn2*^*−/−*^ mESCs relative to WT mESCs. DEGs shared between all three genotypes are highlighted as common. *D*, clustered heatmap of -log2 FC in expression for a combined list of DEGs in *Hmgn1*^*−/−*^ mESCs, *Hmgn2*^*−/−*^ mESCs, and *Hmgn1*^*−/−*^*Hmgn2*^*−/−*^ mESCs, all relative to WT mESCs. *E*, bar graphs of -log2 FC in expression of HMGN genes (*Hmgn1*, *Hmgn2*, *Hmgn3*, *Hmgn4*, and *Hmgn5*), HMGB genes (*Hmgb1*, *Hmgb2*, *Hmgb3*, and *Hmgb4*), and HMGA genes (*Hmga1* and *Hmga2*) in *Hmgn1*^*−/−*^ mESCs, *Hmgn2*^*−/−*^ mESCs, and *Hmgn1*^*−/−*^*Hmgn2*^*−/−*^ mESCs relative to WT mESCs. *Asterisks* indicate significant differences from WT determined using DESeq2 (*p*-adjusted  < 0.01, L_2_FC ≥|1|). *F*, bar graphs of -log2 FC in expression of pluripotency genes (*Pou5f1*, *Sox2*, and *Nanog*), ectodermal lineage genes (*Pax6* and *Nestin*), endodermal lineage genes (*Gata6* and *Sox17*), and mesodermal genes (*Kdr* and *Pdgfra*) in *Hmgn1*^*−/−*^ mESCs, *Hmgn2*^*−/−*^ mESCs, and *Hmgn1*^*−/−*^*Hmgn2*^*−/−*^ mESCs relative to WT mESCs. *Asterisks* indicate significant differences from WT determined using DESeq2 (*p*-adjusted  < 0.01, L_2_FC ≥|1|). *G*, Gene Ontology (GO) analysis for biological processes correlated with DEGs that are upregulated and downregulated in *Hmgn1*^*−/−*^*Hmgn2*^*−/−*^ mESCs relative to WT mESCs. HMGN, High Mobility Nucleosome-binding protein; mESC, mouse embryonic stem cell.

How HMGN1 and HMGN2 proteins regulate gene expression is poorly understood. To investigate the roles of HMGN1 and HMGN2 in mESC gene regulation, CRISPR–Cas9 genome editing was used to generate single knockout cell lines: *Hmgn1*^*−/−*^ mESCs and *Hmgn2*^*−/−*^ mESCs (Fig. 2, *A* and *B*, Fig. S2, *A*–*B*). RNA-Seq was then performed for WT mESCs, *Hmgn1*^*−/−*^ mESCs, and *Hmgn2*^*−/−*^ mESCs, and differential gene expression analysis was performed utilizing DESeq2 (*p*-adjusted  < 0.01, log_2_ fold change [FC] ≥|1|) (Fig. S2*C*). *Hmgn1*^*−/−*^ mESCs displayed 676 differentially expressed genes (DEGs) compared with WT mESCs, and *Hmgn2*^*−/−*^ mESCs displayed 693 DEGs compared with WT mESCs (Table S3). An overlap of the DEGs identified in every single knockout revealed 555 genes that were misexpressed upon loss of either HMGN1 or HMGN2, whereas 121 genes were uniquely misexpressed in *Hmgn1*^*−/−*^ mESCs and 138 genes were uniquely misexpressed in *Hmgn2*^*−/−*^ mESCs (Fig. S2*D*). Importantly, the direction of change of the DEGs identified in *Hmgn1*^*−/−*^ mESCs and *Hmgn2*^*−/−*^ mESCs was strongly positively correlated (*R*^*2*^ = 0.9033), revealing that genes upregulated in one single knockout cell line were also frequently upregulated in the other, and genes that were downregulated in one single knockout line were also frequently downregulated in the other (Fig. S2*E*). A combined list of all DEGs in *Hmgn1*^*−/−*^ mESCs and *Hmgn2*^*−/−*^ mESCs was generated (814 genes), and the –log_2_ FC signal was clustered and plotted, revealing a strikingly similar pattern of gene expression changes in cells lacking HMGN1 or HMGN2 (Fig. S2*F*). Notably, the majority of DEGs in *Hmgn1*^*−/−*^ mESCs and *Hmgn2*^*−/−*^ mESCs were downregulated (Fig. S2*G*), indicating that HMGN1 and HMGN2 play an activating role in gene expression in stem cells.

### Dual loss of HMGN1 and HMGN2 alters stem cell identity gene expression and is similar to the individual loss of HMGN1 or HMGN2

Given the significant overlap of HMGN1- and HMGN2-binding sites in the genome, and the fact that a single HMGN protein can bind to the acidic patch on the nucleosome core at a time, we sought to investigate potential functional redundancy between HMGN1 and HMGN2 by generating an *Hmgn1*^*−/−*^*Hmgn2*^*−/−*^ double knockout mESC line with CRISPR–Cas9 genome editing (Fig. 2, *A* and *B*, Fig. S2, *A*–*B*). RNA-Seq of *Hmgn1*^*−/−*^*Hmgn2*^*−/−*^ mESCs revealed 731 DEGs compared with WT mESCs (*p*-adjusted  < 0.01, log_2_ FC ≥|1|) (Table S3, Fig. S2*C*). Strikingly, dual loss of both HMGN1 and HMGN2 did not cause a major increase in the number of DEGs compared with either single knockout cell line. An overlap of DEGs in *Hmgn1*^*−/−*^ mESCs, *Hmgn2*^*−/−*^ mESCs, and *Hmgn1*^*−/−*^*Hmgn2*^*−/−*^ mESCs revealed 476 genes that were commonly misexpressed upon loss of HMGN1 and/or HMGN2 (Fig. 2*C*). Interestingly, a combined list of all DEGs in *Hmgn1*^*−/−*^ mESCs, *Hmgn2*^*−/−*^ mESCs, *Hmgn1*^*−/−*^*Hmgn2*^*−/−*^ mESCs (954) showed that the –log_2_ FC of DEGs in *Hmgn1*^*−/−*^*Hmgn2*^*−/−*^ mESCs was similar to that in *Hmgn1*^*−/−*^ mESCs and *Hmgn2*^*−/−*^ mESCs (Fig. 2*D*). Genes that were differentially expressed upon loss of HMGN1 were similarly affected by loss of HMGN2. In addition, loss of both HMGN1 and HMGN2 yielded nearly identical gene expression changes to either single knockout cell line. We investigated the extent to which DEGs were bound by HMGN1 and/or HMGN2 in WT mESCs and found that about half of the upregulated genes were bound by HMGN1 and/or HMGN2, and about half of the downregulated genes were bound by HMGN1 and/or HMGN2 (Fig. S2, *H*–*I*).

To examine whether HMGN1, HMGN2, or other HMGN proteins may dynamically compensate for the loss of HMGN1 and/or HMGN2 in either *Hmgn1*^*−/−*^ mESCs, *Hmgn2*^*−/−*^ mESCs, or *Hmgn1*^*−/−*^*Hmgn2*^*−/−*^ mESCs, we measured the transcript levels of all *Hmgn* genes, as well as the other genes of the HMG superfamily. Loss of HMGN1 or HMGN2 did not significantly change the transcript level of the other gene or *Hmgn3*, *Hmgn4*, or *Hmgn5* (Fig. 2*E*). Transcript levels of High Mobility Group HMG-Box (*Hmgb*) genes as well as the gene for High Mobility Group AT-Hook protein 1 (*Hmga1*) were not altered by the loss of HMGN1 and/or HMGN2. However, *Hmga2* transcript levels were significantly decreased upon the loss of HMGN1 and/or HMGN2. This shows that loss of a single HMGN protein does not lead to compensation in the form of increasing transcription of a different *Hmgn* gene and supports the interpretation that HMGN1 and HMGN2 act independently at a common set of genes.

Loss of HMGN1 and/or HMGN2 altered the expression of genes involved in cell differentiation. *Hmgn1*^*−/−*^ mESCs, *Hmgn2*^*−/−*^ mESCs, and *Hmgn1*^*−/−*^*Hmgn2*^*−/−*^ mESCs displayed significant increases in the expression of endoderm genes *Gata6* and *Sox17* as well as increased expression of the mesoderm gene *Pdgfra* (Fig. 2*F*). Gene Ontology analysis of the upregulated and downregulated genes in *Hmgn1*^*−/−*^*Hmgn2*^*−/−*^ mESCs was performed and revealed that upregulated genes were significantly enriched for biological processes like endodermal cell fate differentiation and endothelial cell differentiation (Fig. 2*G*, Table S4). Downregulated genes were significantly enriched in biological processes, such as macrophage proliferation and microglial cell proliferation.

### Cohesin and CTCF localization is not affected by the loss of HMGN1 and HMGN2

**Figure 3:**
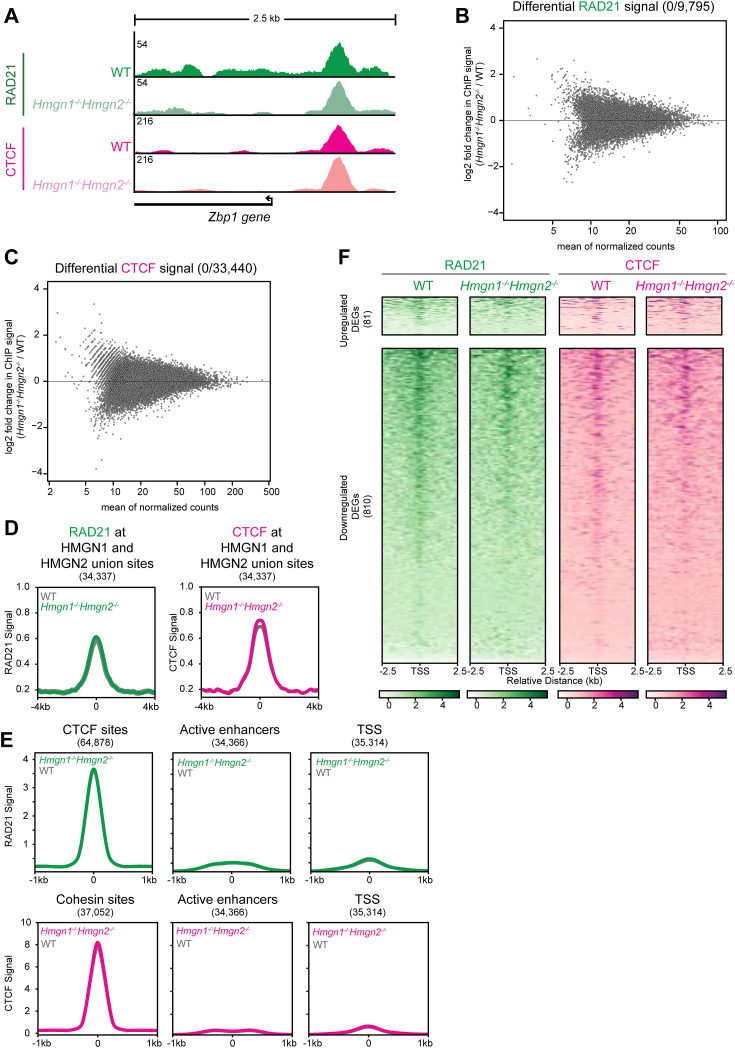
**Cohesin and CTCF localization on chromatin is not dependent on HMGN1 or HMGN2**. *A*, genome browser tracks of RAD21 and CTCF ChIP-Seq signal near the promoter of *Zbp1* (differentially expressed gene in *Hmgn1*^*−/−*^*Hmgn2*^*−/−*^ mESCs) in WT mESCs and *Hmgn1*^*−/−*^*Hmgn2*^*−/−*^ mESCs. *B*, MA plot showing differential enrichment of RAD21 signal between WT mESCs and *Hmgn1*^*−/−*^*Hmgn2*^*−/−*^ mESCs at conserved binding sites. *C*, MA plot showing differential enrichment of CTCF signal between WT mESCs and *Hmgn1*^*−/−*^*Hmgn2*^*−/−*^ mESCs at conserved binding sites. *D*, average signal plots of RAD21 and CTCF ChIP-Seq signal at a union list of all HMGN1 and HMGN2 peaks in WT mESCs and *Hmgn1*^*−/−*^*Hmgn2*^*−/−*^ mESCs (Z-score normalized). *E*, average signal plots of RAD21 and CTCF ChIP-Seq signal in WT mESCs and *Hmgn1*^*−/−*^*Hmgn2*^*−/−*^ mESCs at CTCF sites, cohesin sites, active enhancers, and transcription start sites (TSSs). *F*, ChIP-Seq signal of RAD21 and CTCF in WT mESCs and *Hmgn1*^*−/−*^*Hmgn2*^*−/−*^ mESCs shown at the promoters of upregulated and downregulated differently expressed genes in either *Hmgn1*^*−/−*^ mESCs, *Hmgn2*^*−/−*^ mESCs, or *Hmgn1*^*−/−*^*Hmgn2*^*−/−*^ mESCs. ChIP-Seq, chromatin immunoprecipitation followed by sequencing; HMGN, High Mobility Nucleosome-binding protein; mESC, mouse embryonic stem cell.

Our ChIP-Seq analysis in WT mESCs identified a class of HMGN1- and HMGN2-binding sites that overlap cohesin and CTCF peaks. Indeed, many active enhancers form long-range DNA interactions to their target promoters, which appear to regulate proper gene expression (2, 30, 31, 32). Whether HMGN1 and HMGN2 binding to chromatin might regulate cohesin- and CTCF-mediated DNA loops, and thus gene expression, is unclear. We investigated whether HMGN proteins affect cohesin and CTCF localization by performing ChIP-Seq for RAD21 (core subunit of cohesin) and CTCF in WT mESCs and *Hmgn1*^*−/−*^*Hmgn2*^*−/−*^ mESCs, employing a spike-in for normalization (Table S2). Loss of HMGN1 and HMGN2 did not cause a significant change in the binding pattern or number of RAD21 peaks or CTCF peaks compared with WT mESCs (Fig. 3*A*, Fig. S3, *A*–*D*). A genome-wide analysis of differential cohesin peaks or CTCF peaks was performed using DiffBind and revealed no significantly altered RAD21 or CTCF peaks in *Hmgn1*^*−/−*^*Hmgn2*^*−/−*^ mESCs relative to WT mESCs (Fig. 3, *B* and *C*). RAD21 and CTCF signal at a union list of HMGN1 and HMGN2 peaks also revealed no significant differences in RAD21 or CTCF signal (Fig. 3*D*). RAD21 signal at CTCF sites, active enhancers, and TSSs was strikingly similar between WT mESCs and *Hmgn1*^*−/−*^*Hmgn2*^*−/−*^ mESCs (Fig. 3*E*). Similarly, CTCF signal at cohesin sites, active enhancers, and TSSs were highly similar between WT mESCs and *Hmgn1*^*−/−*^*Hmgn2*^*−/−*^ mESCs (Fig. 3*E*). RAD21 and CTCF signal at the TSSs of upregulated and downregulated DEGs that are found in either *Hmgn1*^*−/−*^ mESCs, *Hmgn2*^*−/−*^ mESCs, or *Hmgn1*^*−/−*^*Hmgn2*^*−/−*^ mESCs reveal strikingly similar signal patterns between WT mESCs and *Hmgn1*^*−/−*^*Hmgn2*^*−/−*^ mESCs, suggesting that cohesin and CTCF localization is not altered at DEGs and does not explain the change in expression of those genes (Fig. 3*F*). These findings indicate that localization of the cohesin complex and CTCF to enhancers, promoters, and insulator sites across the genome is independent of HMGN1 and HMGN2, and therefore, it is unlikely that HMGN proteins contribute to DNA loops and chromosome structure. This also suggests that the changes in gene expression seen in *Hmgn1*^*−/−*^*Hmgn2*^*−/−*^ mESCs are not because of changes in enhancer–promoter loops, but rather, changes in the local chromatin state at enhancers and promoters.

### HMGN1 and HMGN2 binding to chromatin is enhanced by histone acetylation and H2A.Z

The conserved N-terminal region of HMGN proteins, termed the NBD, binds to the acidic patch on the nucleosome core “disk face” formed by H2A–H2B (17, 18, 33). Our genome-wide binding analysis shows that HMGN proteins localize to active chromatin marked by H3K27ac and H3K4me3, yet HMGN proteins lack domains for reading the underlying DNA sequence or for reading the presence of histone PTMs. It has recently been suggested that modifications to histone tails can alter their positioning relative to the nucleosome core (34, 35). For instance, H3K4 readers, such as MLL1 and MLL4, have been shown to have enhanced binding to, and greater catalytic activity on, H3 tails with individual or combinatorial acetylation at K9ac, K14ac, K18ac, and K23ac (36, 37). These results indicate that unmodified histone tails may adopt a collapsed state where they make contacts with the nucleosome core and negatively charged DNA, whereas acetylation on histone tails neutralizes the positive charge of the lysine residues and releases the H3 tail from nucleosomal DNA, allowing them to extend away from the nucleosome core and become accessible substrates for histone reader/writer/eraser proteins and complexes (38).

**Figure 4:**
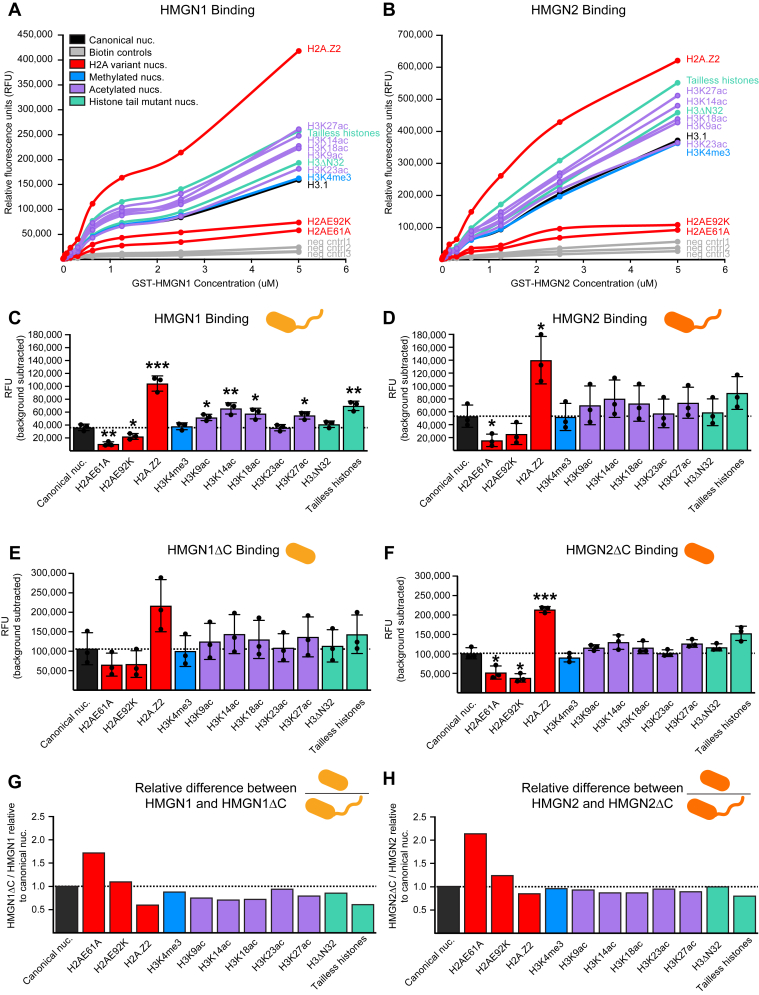
**HMGN1 and HMGN2 preferentially bind to nucleosomes containing H2A.Z and acetylated histone tails**. *A*, titration of GST-HMGN1 protein with each nucleosome-bead conjugate, expressed as relative fluorescence units before normalization. *B*, titration of GST-HMGN2 protein with each nucleosome-bead conjugate, expressed as relative fluorescence units before normalization. One outlier data point was excluded from the H2A.Z variant at the 5 nM protein concentration. *C*, GST-HMGN1 binding relative to the canonical nucleosome with background subtracted. Background signal captured by negative control bead conjugates (average signal of 50 mM BSA-bead, 100 mM BSA-bead, and 200 mM BSA-bead conjugates) and wells containing 0 mM GST-HMGN1 protein were subtracted from raw values for each nucleosome-bead conjugate at 0.625 nM GST-HMGN1 concentration. A *t* test was used to assess statistical significance, with one *asterisk* (∗) denoting a *p* value less than 0.05, ∗∗ indicating a *p* value less than 0.01, and ∗∗∗ representing a *p* value less than 0.001. Error bars represent the standard deviation calculated from three technical replicates. *D*, GST-HMGN2 binding relative to the canonical nucleosome with background subtracted. Background signal captured by negative control bead conjugates (average signal of 50 mM BSA-bead, 100 mM BSA-bead, and 200 mM BSA-bead conjugates) and wells containing 0 mM GST-HMGN2 protein were subtracted from raw values for each nucleosome-bead conjugate at 0.625 nM GST-HMGN2 concentration. A *t* test was used to assess statistical significance, with one *asterisk* (∗) denoting a *p* value less than 0.05, ∗∗ indicating a *p* value less than 0.01, and ∗∗∗ representing a *p* value less than 0.001. Error bars represent the standard deviation calculated from three technical replicates. *E*, GST-HMGN1ΔC binding relative to the canonical nucleosome with background subtracted. Background signal captured by negative control bead conjugates (average signal of 50 mM BSA-bead, 100 mM BSA-bead, and 200 mM BSA-bead conjugates) and wells containing 0 mM GST-HMGN1ΔC protein were subtracted from raw values for each nucleosome-bead conjugate at 0.625 nM GST-HMGN1ΔC concentration. A *t* test was used to assess statistical significance, with one *asterisk* (∗) denoting a *p* value less than 0.05, ∗∗ indicating a *p* value less than 0.01, and ∗∗∗ representing a *p* value less than 0.001. Error bars represent the standard deviation calculated from three technical replicates. *F*, GST-HMGN2ΔC binding relative to the canonical nucleosome with background subtracted. Background signal captured by negative control bead conjugates (average signal of 50 mM BSA-bead, 100 mM BSA-bead, and 200 mM BSA-bead conjugates) and wells containing 0 mM GST-HMGN2ΔC protein were subtracted from raw values for each nucleosome-bead conjugate at 0.625 nM GST-HMGN2ΔC concentration. A *t* test was used to assess statistical significance, with one *asterisk* (∗) denoting a *p* value less than 0.05, ∗∗ indicating a *p* value less than 0.01, and ∗∗∗ representing a *p* value less than 0.001. Error bars represent the standard deviation calculated from three technical replicates. *G*, bar graph of normalized GST-HMGN1ΔC nucleosome binding data over GST-HMGN1 nucleosome binding data relative to unmodified H3.1 mononucleosome-bead conjugate. *H*, bar graph of normalized GST-HMGN2ΔC nucleosome binding data over GST-HMGN2 nucleosome binding data relative to unmodified H3.1 mononucleosome-bead conjugate. BSA, bovine serum albumin; GST, glutathione-*S*-transferase; HMGN, High Mobility Nucleosome-binding protein.

To investigate the extent to which histone tail PTMs and/or histone variants regulate binding of HMGN proteins to nucleosomes, we expressed and purified human HMGN proteins in *Escherichia coli* and employed a high-performance *in vitro* multiplex platform to measure the binding of glutathione-*S*-transferase (GST)-HMGN1 and GST-HMGN2 to nucleosomes harboring individual histone tail PTMs, histone variants, or histone mutants (39). The results showed that HMGN1 and HMGN2 bind preferentially to nucleosomes containing H2A.Z and to those containing histone tail acetylation (H3K9ac, H3K14ac, H3K18ac, and H3K27ac) (Fig. 4, *A*–*D*, Table S5). It is important to highlight that HMGN1 and HMGN2 still show some level of binding to canonical unmodified nucleosomes, and the results are depicted to emphasize the enhanced or diminished ability of HMGN1 and HMGN2 to bind to nucleosomes with modifications in comparison to the canonical nucleosome. Strikingly, we find that HMGN1 and HMGN2 show the strongest preference for nucleosomes containing the H2A.Z histone variant. H2A.Z nucleosomes differ from canonical H2A nucleosomes in that H2A.Z has an extended acidic patch compared with canonical H2A, and H2A.Z histones have shorter C-terminal tails (residues 123–128) compared with canonical H2A C-terminal tails (residues 121–130) (40). Importantly, HMGN1 and HMGN2 binding to nucleosomes harboring single-point mutations within the H2A acidic patch (H2AE61A and H2AE92K) was significantly decreased compared with unmodified nucleosomes containing canonical WT H2A, indicating that a single-point mutation to the H2A acidic patch drastically disrupts the ability of HMGN proteins to bind to nucleosomes. HMGN1 and HMGN2 bound to methylated nucleosomes (H3K4me3, H3K9me3, H3K27me3, and H3K36me3) and nucleosomes containing unmodified H3 to the same extent (Fig. S4, *A*–*D*). These results demonstrate that the primary determinant for HMGN1 and HMGN2 binding to nucleosomes is the interface between the HMGN NBD and the acidic patch on the nucleosome face. Secondarily, acetylation of the H3 tail enhances HMGN1 and HMGN2 binding to the nucleosome. This likely occurs by promoting an extended conformation of the histone tails, which may (1) increase exposure of the acidic patch for HMGN binding; (2) facilitate the interaction between lysine residues in the HMGN NBD and DNA near the nucleosome entry/exit site; and/or (3) enable the HMGN C-terminal tail to engage the acetylated H3 tail.

Recent work suggests that unmodified histone tails exist in a collapsed state on the globular nucleosome core and shield the nucleosome from chromatin-associating proteins (41, 42, 43). To investigate the extent to which histone tails regulate binding of HMGN proteins to the acidic patch on H2A and H2B, we performed binding assays using nucleosomes lacking the H3 tail or all eight histone tails. The results showed that HMGN1 and HMGN2 display enhanced binding to nucleosomes lacking all eight histone tails compared with WT unmodified nucleosomes that contain histone tails (Fig. 4, *A*–*D*). The binding of HMGN1 and HMGN2 to nucleosomes lacking H3 tails was similar to unmodified nucleosomes. These results are consistent with a model in which unmodified histone tails exist in a collapsed state and at least partially occlude the H2A–H2B acidic patch on the nucleosome disk face, thus blocking interactions with chromatin-associating proteins (42).

Several studies have suggested that the C terminus of HMGN proteins may reside in physical proximity to the histone H3 tail, near linker DNA, and the nucleosome dyad axis (17, 18, 44). To investigate the extent to which the unstructured C-terminal tail of HMGN proteins contributes to HMGN binding to nucleosomes, we measured the binding of GST-HMGN1 and GST-HMGN2 lacking the C terminus (termed HMGN1ΔC and HMGN2ΔC) to nucleosomes harboring individual histone tail PTMs. The results showed that HMGN1ΔC and HMGN2ΔC displayed the same trend of preferential binding to acetylated nucleosomes and H2A.Z-containing nucleosomes as full-length HMGN1 and HMGN2 (Fig. 4, *E* and *F*, Fig. S4, *E*–*G*). In addition, the relative binding of truncated HMGN proteins to acetylated nucleosomes and H2A.Z-containing nucleosomes was lower than that of full-length HMGN proteins (Fig. 4, *G* and *H*). These results suggest that the C terminus of HMGN proteins is a minor, secondary contributor to the binding of HMGN proteins to nucleosomes, after the primary interaction of the HMGN NBD to the H2A–H2B acidic patch, and that the HMGN C terminus does not confer specificity for nucleosomal substrates. Together, these results indicate that full-length HMGN proteins that contain an NBD and a C-terminal unstructured region bind preferentially to nucleosomal substrates that contain H2A.Z and H3 tail acetylation, which are hallmarks of transcriptionally active chromatin regions.

### HMGN1 and HMGN2 limit p300-mediated histone acetylation

**Figure 5:**
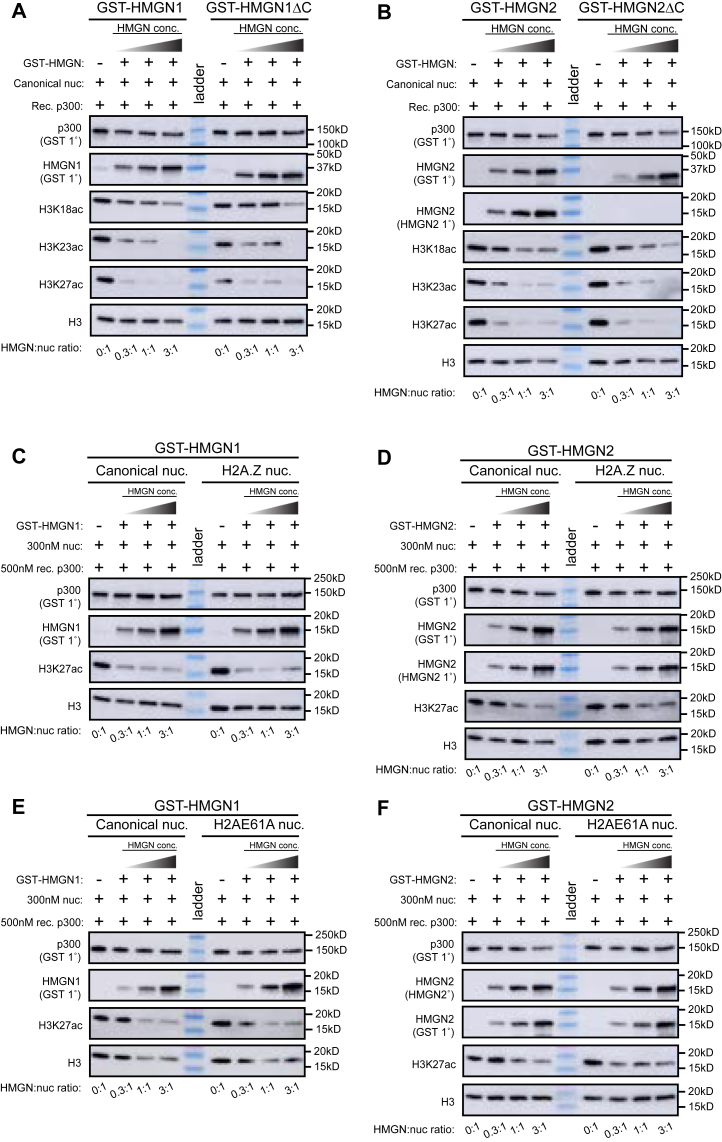
**HMGN1 and HMGN2 reduce p300-mediated acetylation of the H3 tail**. *A*, Western blot analysis of HAT reaction mixtures containing equal amounts of recombinant mononucleosomes (canonical nuc.) with 147 base pairs of 601 sequence DNA, preincubated with variable amounts of recombinant GST-HMGN1 or GST-HMGN1ΔC protein and then incubated with equal amounts of recombinant p300 and acetyl-CoA. H3 lysine acetylation of K18, K23, and K27 was imaged *via* PTM-specific antibodies. *B*, Western blot analysis of HAT reaction mixtures containing equal amounts of recombinant mononucleosomes (canonical nuc.) with 147 base pairs of 601 sequence DNA, preincubated with variable amounts of recombinant GST-HMGN2 or GST-HMGN2ΔC protein and then incubated with equal amounts of recombinant p300 and acetyl-CoA. H3 lysine acetylation of K18, K23, and K27 was imaged *via* PTM-specific antibodies. *C*, Western blot analysis of HAT reaction mixtures containing equal amounts of recombinant mononucleosomes (canonical nuc.) or recombinant H2A.Z-containing mononucleosomes with 147 base pairs of 601 sequence DNA, preincubated with variable amounts of recombinant GST-HMGN1 protein and then incubated with equal amounts of recombinant p300 and acetyl-CoA. H3 lysine acetylation of K27 was imaged *via* PTM-specific antibodies. *D*, Western blot analysis of HAT reaction mixtures containing equal amounts of recombinant mononucleosomes (canonical nuc.) or recombinant H2A.Z-containing mononucleosomes with 147 base pairs of 601 sequence DNA, preincubated with variable amounts of recombinant GST-HMGN2 protein and then incubated with equal amounts of recombinant p300 and acetyl-CoA. H3 lysine acetylation of K27 was imaged *via* PTM-specific antibodies. *E*, Western blot analysis of HAT reaction mixtures containing equal amounts of recombinant mononucleosomes (canonical nuc.) or recombinant H2AE61A mononucleosomes with 147 base pairs of 601 sequence DNA, preincubated with variable amounts of recombinant GST-HMGN1 protein and then incubated with equal amounts of recombinant p300 and acetyl-CoA. H3 lysine acetylation of K27 was imaged *via* PTM-specific antibodies. *F*, Western blot analysis of HAT reaction mixtures containing equal amounts of recombinant mononucleosomes (canonical nuc.) or recombinant H2AE61A mononucleosomes with 147 base pairs of 601 sequence DNA, preincubated with variable amounts of recombinant GST-HMGN2 protein and then incubated with equal amounts of recombinant p300 and acetyl-CoA. H3 lysine acetylation of K27 was imaged *via* PTM-specific antibodies. GST, glutathione-*S*-transferase; HAT, histone acetyltransferase; HMGN, High Mobility Nucleosome-binding protein; PTM, post-translational modification.

How HMGN1 and HMGN2 binding to nucleosomes at active enhancers and promoters alters chromatin state and gene expression is unclear. The nucleosome acidic patch is a key interface used by many chromatin-binding proteins (16). Previous studies have found that HMGN proteins modulate p300/CBP-associated factor–mediated acetylation of the H3 tail, suggesting that HMGN proteins influence the activity of writer enzymes (25, 45, 46, 47). We hypothesized that the binding of HMGN1 and HMGN2 to nucleosomes could alter the ability of other chromatin-associating proteins to bind the acidic patch or engage histone tails. To understand whether HMGN proteins influence the activity of chromatin-modifying enzymes on nucleosomal substrates, we performed *in vitro* histone acetyltransferase (HAT) assays to investigate the ability of recombinant p300 catalytic core to acetylate nucleosomes prebound by HMGN proteins. We focused on p300, as this enzyme is well known to acetylate promoter and enhancer nucleosomes where HMGN proteins are found. To perform this assay, GST-HMGN1 or GST-HMGN2 proteins were first incubated with unmodified canonical mononucleosomes to generate HMGN–nucleosome complexes. Then recombinant p300 and acetyl-CoA were added, and the acetylation of H3 lysine residues was assayed by immunoblotting. The results show that HMGN1 or HMGN2 binding to nucleosomes decreased p300-mediated acetylation of the H3 lysine residues H3K18ac, H3K23ac, and H3K27ac (Fig. 5, *A* and *B*, Fig. S5, *A*–*D*). Importantly, the reduction of p300-mediated histone acetylation was observed at various stoichiometric ratios of HMGN protein to nucleosome: 0.3:1, 1:1, and 3:1 (HMGN:nuc). HMGN1 reduced the levels of acetylation mediated by p300 on H3K18, H3K23, and H3K27 (Fig. 5*A*, Fig. S5, *A*–*B*). HMGN2 also reduced the levels of H3K18ac, H3K23ac, and H3K27ac (Fig. 5*B*, Fig. S5, *C*–*D*). These results indicate that p300 binding and/or catalytic activity on HMGN–nucleosome substrates is reduced compared with nucleosomes that lack HMGN proteins.

### The HMGN C-terminal tail does not regulate p300-mediated histone tail acetylation

Cryo-EM structures of the p300 catalytic core bound to a nucleosome suggest that p300 can adopt multiple conformations/states, and during its acetylation of histones, p300 directly interacts with the H3 tail and does not contact the nucleosome acidic patch (48). We therefore sought to test whether the HMGN C-terminal region might interact with the H3 tail and affect p300-mediated acetylation of H3 tail residues. To ask this question, we again performed HAT assays with GST-HMGN1ΔC and GST-HMGN2ΔC proteins and found that both truncated HMGN proteins limited p300-mediated histone acetylation of H3K18ac, H3K23ac, and H3K27ac to a similar extent as full-length HMGN proteins (Fig. 5, *A* and *B*). Importantly, the reduction of p300-mediated histone acetylation was observed at various stoichiometric ratios of HMGN protein to nucleosome: 0.3:1, 1:1, and 3:1 (HMGN:nuc). These results suggest that the C-terminal tail of HMGN proteins does not have a strong effect on p300-mediated acetylation of H3 tail residues.

To determine whether the HMGN-mediated reduction of histone acetylation is influenced by nucleosome composition, we next performed the *in vitro* acetyltransferase assays using nucleosomes containing the histone variant H2A.Z or the H2AE61A acidic patch mutation. GST-HMGN1 and GST-HMGN2 were preincubated with canonical or H2A.Z-containing nucleosomes, before addition of recombinant p300 and acetyl-CoA, and immunoblotting was performed to assess acetylation of H3K27. Across all stoichiometries tested, we observed similar decreases in p300-mediated acetylation on canonical and H2A.Z-containing nucleosomes (Fig. 5, *C* and *D*). Therefore, the twofold binding preference of HMGN to H2A.Z-containing nucleosomes over canonical nucleosomes did not result in a corresponding decrease in p300 activity under the conditions of the assay. Similarly, despite the reduced binding of HMGN proteins to H2AE61A-containing nucleosomes, we observed a similar level of p300-mediated acetylation on acidic patch mutant nucleosomes relative to canonical nucleosomes, under these assay conditions (Fig. 5, *E* and *F*).

### HMGN1 and HMGN2 do not alter the *in vivo* recovery kinetics of p300-mediated histone acetylation

To investigate the role of HMGN1 and HMGN2 on histone acetylation *in vivo*, we first treated cells with a p300 inhibitor to reduce histone acetylation and then measured acetylation recovery after washout of the inhibitor. WT mESCs and *Hmgn1*^*−/−*^*Hmgn2*^*−/−*^ mESCs were treated with either dimethyl sulfoxide (DMSO) or 10 μM A-485 (p300 inhibitor) for 1 h. After the 1 h treatment, cells were immediately collected at the 0 min recovery time point, or a media change was performed to remove the p300 inhibitor. Cells were allowed to recover for 10, 30, 60, or 120 min post-inhibitor washout. The results show that loss of both HMGN1 and HMGN2 did not alter the rate of deposition of histone acetylation *in vivo* (Fig. S6, *A*–*E*). It is possible that other HATs or histone deacetylases (HDACs) compensate for the loss of p300 activity *in vivo*. These results suggest a complex and dynamic relationship between HMGN proteins, HATs, and HDACs *in vivo* and that disruption of any one of these factors may trigger compensatory processes that maintain normal histone acetylation levels *in vivo*.

### Loss of HMGN1 and HMGN2 increases steady-state H3K27me2 and H3K27me3

**Figure 6:**
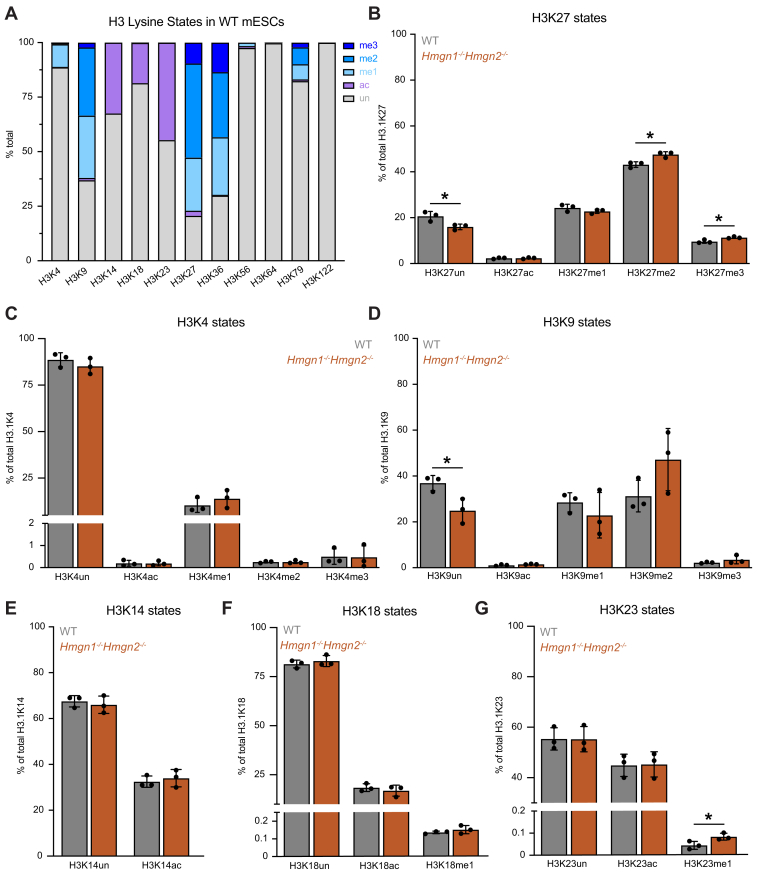
**Loss of HMGN1 and HMGN2 increases steady-state H3K27me2/3**. *A*, stacked bar chart showing the relative abundance of different modification states for histone H3 lysine residues in WT mESCs. Colors indicate modification types: trimethylated (*dark blue*), dimethylated (*medium blue*), monomethylated (*light blue*), acetylated (*purple*), and unmodified (*gray*). *B*, bar graph showing the relative abundance of unmodified, acetylated, and methylated H3K27 states in WT (*gray*) and *Hmgn1*^*−/−*^*Hmgn2*^*−/−*^ (*dark orange*) mESCs. Values represent the percentage of total H3.1K27 in each modification state (mean ± SD, n = 3 biological replicates). Loss of HMGN1 and HMGN2 results in a significant decrease in unmodified H3K27, accompanied by an increase in H3K27me2 and H3K27me3 (*p* < 0.05, Student’s *t* test). *C*, bar graph showing the relative abundance of unmodified, acetylated, and methylated H3K4 states in WT (*gray*) and *Hmgn1*^*−/−*^*Hmgn2*^*−/−*^ (*dark orange*) mESCs. Values represent the percentage of total H3.1K4 in each modification state (mean ± SD, n = 3 biological replicates). *D*, bar graph showing the relative abundance of unmodified, acetylated, and methylated H3K9 states in WT (*gray*) and *Hmgn1*^*−/−*^*Hmgn2*^*−/−*^ (*dark orange*) mESCs. Values represent the percentage of total H3.1K9 in each modification state (mean ± SD, n = 3 biological replicates). Loss of HMGN1 and HMGN2 results in a significant decrease in unmodified H3K9 (*p* < 0.05, Student’s *t* test). *E*, bar graph showing the relative abundance of unmodified and acetylated H3K14 states in WT (*gray*) and *Hmgn1*^*−/−*^*Hmgn2*^*−/−*^ (*dark orange*) mESCs. Values represent the percentage of total H3.1K4 in each modification state (mean ± SD, n = 3). *F*, bar graph showing the relative abundance of unmodified, acetylated, and methylated H3K18 states in WT (*gray*) and *Hmgn1*^*−/−*^*Hmgn2*^*−/−*^ (*dark orange*) mESCs. Values represent the percentage of total H3.1K18 in each modification state (mean ± SD, n = 3 biological replicates). *G*, bar graph showing the relative abundance of unmodified, acetylated, and methylated H3K23 states in WT (*gray*) and *Hmgn1*^*−/−*^*Hmgn2*^*−/−*^ (*dark orange*) mESCs. Values represent the percentage of total H3.1K23 in each modification state (mean ± SD, n = 3 biological replicates). Loss of HMGN1 and HMGN2 results in a significant increase in H3K23me1 (*p* < 0.05, Student’s *t* test). HMGN, High Mobility Nucleosome-binding protein; mESC, mouse embryonic stem cell.

To quantify the levels of specific histone modifications in WT and *Hmgn1*^*−/−*^*Hmgn2*^*−/−*^ mESCs, we performed mass spectrometry–based quantification of a panel of histone residue states (Table S6). The H3 tail displays a highly diverse distribution of modification states for different residues in WT mESCs (Fig. 6*A*). Most H3 lysine residues predominantly exist in the unmodified state, although several exhibited frequent modifications. H3K4, H3K56, H3K64, H3K79, and H3K122 often exist in the unmodified state. H3K9, H3K27, and H3K36 were predominantly methylated, with these residues existing in monomethylated, dimethylated, or trimethylated forms roughly 70% of the time. H3K14, H3K18, and H3K23 were often acetylated, accounting for 15% to 45% of their states. These data establish a baseline of histone H3 lysine modification states in WT cells.

*Hmgn1*^*−/−*^*Hmgn2*^*−/−*^ mESCs showed changes in the modification state of H3K27 with a reduction in the unmodified state and an increase in H3K27me2 and H3K27me3 (*p* < 0.05) relative to WT mESCs (Fig. 6*B*, Fig. S7). Levels of H3K27ac and H3K27me1 were largely unchanged in *Hmgn1*^*−/−*^*Hmgn2*^*−/−*^ cells relative to WT (Fig. 6*B*). These results indicate that HMGN1 and HMGN2 help maintain proper H3K27 methylation homeostasis, potentially by influencing the activity, recruitment, or accessibility of PRC2 or H3K27 demethylases *in vivo*.

In contrast, H3K4 (Fig. 6*C*) displayed no significant difference in modification states between WT and *Hmgn1*^*−/−*^*Hmgn2*^*−/−*^ cells, indicating that HMGN1 and HMGN2 loss does not broadly alter methylation or acetylation across all lysines. At H3K9 (Fig. 6*D*), *Hmgn1*^*−/−*^*Hmgn2*^*−/−*^ cells showed a decrease in the unmodified state without a corresponding increase in any measured modification (ac, me1, me2, or me3). H3K14 and H3K18 showed no detectable changes in their modification profiles (Fig. 6, *E* and *F*). Finally, at H3K23 (Fig. 6*G*), there was an apparent increase in H3K23me1 levels in *Hmgn1*^*−/−*^*Hmgn2*^*−/−*^ mESCs, though without a significant decrease in the unmodified fraction. Overall, these findings indicate that HMGN1 and HMGN2 selectively affect specific H3 modification sites, most prominently H3K27, while leaving others largely unchanged, suggesting a specific role for HMGN proteins in sensing and regulating, perhaps directly and indirectly, histone post-translational modification (PTM) homeostasis in cells.

## Discussion

In this study, we investigated the role of HMGN proteins in shaping the chromatin landscape defined by histone PTMs. How active chromatin regions acquire H2A.Z, histone acetylation modifications, and binding of specific chromatin-associating proteins that promote transcription is unclear. To interrogate the role of HMGN proteins in establishing and maintaining the active chromatin state, we sought to examine their roles in nucleosome binding, steady-state histone modifications, and gene expression. Nucleosome-binding assays reveal key determinants of HMGN binding, including the nucleosome acidic patch and histone tail modifications. We present the first evidence that HMGN proteins preferentially bind nucleosomes containing the H2A.Z variant, which occupies actively transcribed promoters and enhancers. This preference is likely because of the extended acidic patch present in H2A.Z histones compared with canonical H2A histones. In addition, active regions of the genome contain nucleosomes that harbor acetylation on numerous H3 lysine residues. We show that HMGN1 and HMGN2 preferentially bind to nucleosomes harboring the singular acetylation marks H3K9ac, H3K14ac, H3K18ac, or H3K27ac. The acetylation of lysine residues neutralizes electrostatic interactions between the lysine residues on the H3 tail and DNA, likely leading to the H3 tail adopting a conformation that extends away from the nucleosome core. Nucleosomes with extended histone tails may serve as better substrates for HMGN binding because of (1) increased exposure of the acidic patch for HMGN binding; (2) availability of the acetylated H3 tail to engage the HMGN C-terminal tail, contributing to multivalent interactions; and/or (3) increased exposure of DNA near the nucleosome entry/exit site for HMGN binding. Consistent with this, we find that HMGN proteins preferentially bind to nucleosomes that lack all histone tails compared with canonical nucleosomes. Finally, we show that the C-terminal tail of HMGN proteins largely does not confer specificity for nucleosomes harboring individual acetylated histone PTMs. These findings provide new insights into how HMGN proteins are recruited to active chromatin, underscoring the role of acetylated histones and H2A.Z-containing nucleosomes as substrates for chromatin-associating proteins. Notably, the involvement of H2A.Z adds to growing evidence that this histone variant plays a multifaceted role in transcriptional regulation. While H2A.Z has traditionally been linked to both gene activation and repression, our data suggest that it may also function as a platform for the selective recruitment of chromatin-associating proteins like HMGNs (49). This expands our understanding of the contribution of H2A.Z to chromatin dynamics beyond its established roles in nucleosome stability and promoter accessibility.

Notably, our findings are somewhat different from prior studies in the field that utilized nucleosomes with 160 bp of DNA, whereas our study employed nucleosomes with 147 bp of DNA (20). Furthermore, the prior work did not detect preferential binding of HMGN proteins to nucleosomes with the individual histone PTMs H3K9ac, H3K14ac, and H3K18ac relative to unmodified H3. Since HMGN proteins contain lysine residues that can interact with DNA, the 160-bp DNA substrates may have allowed for nonspecific binding that dampened the signal of preferential nucleosome binding observed on 147 bp nucleosomes. In addition, our use of a multiplexed, highly sensitive assay may provide enhanced power to detect relatively small but significant differences in binding. We propose a model in which the several individual features of nucleosomes that each enhance HMGN binding to nucleosomes individually may act synergistically to promote robust binding of HMGN proteins to active chromatin that often harbors combinations of these individual features within the cells.

Our study examines the extent to which HMGN proteins influence DNA loops and chromosome structure. We find that HMGNs do not affect the genome-wide localization of cohesin or CTCF; therefore, we conclude that HMGN proteins are unlikely to directly affect enhancer–promoter looping. This is consistent with a study that investigated the role of HMGN proteins in mediating the spatial organization of the genome with regard to spatial segregation of euchromatin (A compartments) and heterochromatin (B compartments), which are generally described as transcriptionally active and inactive regions of the genome, respectively (29). HMGN proteins were found to be primarily localized within A compartments, and loss of HMGN proteins did not significantly impact genome-wide compartmentalization into A and B compartments. Both studies indicate that the role of HMGN proteins in chromatin dynamics does not involve cohesin- and CTCF-mediated chromosome structure but rather is related to chromatin dynamics and binding of chromatin-associating proteins.

Our findings reveal that HMGN proteins reduce p300 activity on specific lysine residues (H3K18, H3K23, and H3K27) on the H3 tail. We find that HMGN proteins have the strongest effect on limiting p300-mediated acetylation of the H3K27 residue. This effect may be caused by the HMGN protein occluding H3K27 more than H3K18 and H3K23 when nucleosome bound. We additionally show that the C-terminal tail of HMGN proteins is largely dispensable for the HMGN-mediated decrease in p300 activity on histone H3 tails, suggesting that the NBD of HMGNs is sufficient to regulate p300-mediated acetylation of H3 tail residues. Surprisingly, the reduction in p300 activity was similar across canonical nucleosomes, H2A.Z-containing nucleosomes, and H2AE61A acidic patch mutant nucleosomes despite differences in HMGN binding affinity to these substrates. This discrepancy likely reflects the different sensitivity of these assays: binding assays quantify physical association between HMGNs and nucleosomes, whereas acetylation assays measure a functional outcome in a defined biochemical system. Thus, increased binding affinity does not necessarily correlate with enhanced inhibition of p300 activity in this *in vitro* acetyltransferase assay. Together, our nucleosome binding data and the HAT data indicate that HMGNs are both sensitive to the acetylation state of nucleosomes and also modulators of the histone PTM landscape.

Notably, our mass spectrometry–based quantification of histone H3 modification states did not detect a global reduction in H3K27ac levels *in vivo*, seemingly contradictory to the strong reduction in H3K27ac observed in our *in vitro* acetyltransferase assays. It is likely that compensation from other HATs or the balance with HDAC activity *in vivo* allows for steady-state H3K27 acetylation levels to be maintained. Notably, previous studies reported changes in specific histone PTM levels between WT and HMGN knockout cells that were not confirmed by our epiproteomic mass spectrometry analysis (28, 46, 50). Specifically, *Hmgn1*^*−/−*^ mouse embryonic fibroblasts were reported to have increased H3S10ph, H3K9ac, and H3K14ac, as well as decreased H3K14ac levels, according to Western blot analysis of extracts (46, 50). The seeming discrepancy between our results and previous reports may arise from the different sensitivities of the assays employed: Western blotting is semiquantitative and can be influenced by antibody specificity and epitope accessibility, whereas mass spectrometry provides a more unbiased and quantitative analysis of multiple PTMs simultaneously. Together, these findings highlight the need for more precise and integrative approaches to accurately measure the impact of HMGN proteins on the histone PTM landscape.

Our mass spectrometry–based analysis of histone H3 modifications in WT mESCs revealed that H3K27 exists predominantly in methylated forms, and *Hmgn1*^*−/−*^*Hmgn2*^*−/−*^ cells have increased H3K27me2 and H3K27me3 levels compared with WT mESCs. Given that H3K27 is already highly methylated under basal conditions, detecting a further significant increase is striking and suggests that HMGN proteins may play an unappreciated role in limiting PRC2 activity through direct and/or indirect mechanisms. The increase in H3K27me2/3 in *Hmgn1*^*−/−*^*Hmgn2*^*−/−*^ cells could reflect enhanced PRC2 catalytic activity, enhanced recruitment or retention of PRC2 at chromatin, or greater nucleosome accessibility for methylation in the absence of HMGNs.

These findings suggest that HMGNs may regulate histone acetylation and methylation by directly modulating the recruitment and/or activity of chromatin-modifying enzymes or indirectly shaping the chromatin environment in ways that affect the availability of histone substrates. We show here that HMGN proteins can modulate p300 acetyltransferase activity and restrict the deposition of repressive H3K27 methylation by PRC2, thereby helping to maintain a dynamic balance between active and repressive chromatin states. Taken together, our data reveal that HMGNs function both as sensors of the chromatin modification landscape and as active modulators of histone PTMs. Our results are consistent with a model whereby nucleosomes initially acquire low levels of acetylation from p300 and/or other HATs, which in turn promotes HMGN recruitment to these sites. Once bound, HMGN proteins limit further p300-mediated acetylation on these nucleosomal substrates, potentially by blocking HAT access. Limiting additional H3 acetylation may be important in cells. Hoffman *et al*. (51) recently reported that RNA polymerase II recruits HDACs to promote active deacetylation of histones at active promoters. Therefore, the nucleosomes at actively transcribed promoters experience a highly dynamic cycle of histone acetylation and deacetylation. HMGN proteins may affect the dynamic balance between HATs and HDACs to maintain and regulate steady-state levels of histone acetylation at active genes. Thus, our *in vivo* and *in vitro* results suggest that HMGNs function both as sensors of the acetylation landscape and as regulators of the histone PTM landscape. This dual role in chromatin state regulation allows HMGNs to fine-tune gene expression programs by coordinating the interplay between active and repressive chromatin states at genes.

## Experimental procedures

### Cell culture

Male v6.5 mESCs were grown under standard mESC conditions as previously described (52). Cells were plated on 0.2% gelatinized tissue culture plates in media containing Knockout Dulbecco’s modified Eagle’s medium (Gibco; catalog no.: 10829-018), 15% fetal bovine serum (VWR; catalog no.: 97068-085), 1000 U/ml LIF (ESGRO; catalog no.: ESG1106), 100 μM nonessential amino acids (Thermo Fisher Scientific; catalog no.: 11140050), 1X GlutaMAX (Thermo Fisher Scientific; catalog no.: 35050-061), 100 U/ml penicillin, 100 μg/ml streptomycin (Thermo Fisher Scientific; catalog no.: 15140-122), and 8 nl/ml of 2-mercaptoethanol (Thermo Fisher Scientific; catalog no.: 21985023). Human embryonic kidney 293T cells (female) were grown on 0.2% gelatinized tissue culture plates with media containing Dulbecco’s modified Eagle’s medium (Gibco; catalog no.: 11995065) supplemented with 10% bovine calf serum (Seradigm; catalog no.: 2100-500), 1X GlutaMAX (Thermo Fisher Scientific; catalog no.: 35050-061), 100 U/ml penicillin, and 100 μg/ml streptomycin (Thermo Fisher Scientific; catalog no.: 15140-122).

### A-485 treatment

Cells treated with the A-485 p300 inhibitor (MedChemExpress; catalog no.: HY-107455) were treated with standard mESC media with a final concentration of 10 μM A-485 for 1 h at 37 °C. After treatment, cells were collected *via* trypsin (Gibco; catalog no.: 12604-013) following the standard protocol outlined above under cell culture.

### Plasmid generation

For single guide RNA (sgRNA) plasmids used for genome editing, the pX330 backbone containing SpCas9 and mCherry genes (AddGene; catalog no.: 42230) was utilized to clone unique sgRNAs targeting the gene of interest. For GST-tagged HMGN plasmids used for protein purification, the pGEX-6P-1 backbone containing the tac promoter, N-terminal GST tag, and the ampicillin resistance gene (VWR; catalog no.: 27-4597-01) was utilized to clone the coding sequence of the gene of interest. Various cloning techniques were employed in plasmid generation, such as Gibson Assembly utilizing the Gibson Assembly Cloning kit (NEB; catalog no.: E5510S) and site-directed mutagenesis utilizing the KLD Enzyme Mix (NEB; catalog no.: M0554S). Primers used for all cloning experiments are included in Table S7.

### Genome editing

To generate single knockouts, WT mESCs were transfected with a sgRNA-containing plasmid *via* Lipofectamine 2000 (Thermo Fisher Scientific; catalog no.: 11-688-027) to induce an INDEL in an early exon of the gene of interest. Twenty-four hours post transfection, cells were inspected for transfection efficiency (aiming for ∼20–50%) *via* visualization of fluorescent markers on the plasmid. After confirming good transfection efficiency, 12,000 cells were seeded onto a prewashed (three times in 3 ml warm PBS incubations) CytoSort Array (Cell Microsystems; catalog no.: CS200S) in a dropwise fashion. Using a CellRaft AIR System (Cell Microsystems), images of the CytoSort array were taken every ∼24 h up to 3-day post transfection to select single-cell fluorescent cells. Cells were collected, expanded, screened for INDELs at the cut site within the gene of interest *via* PCR and Sanger sequencing, and cryogenically stored. Knockouts were determined *via* RT–quantitative PCR (qPCR) and Western blotting for the protein of interest. The sgRNA sequences used for *Hmgn1* and *Hmgn2* genome editing are included in Table S7, and the edited sequences are depicted in Figure S2, *A*–*B*.

*Hmgn1*^−/−^ mESCs contain a homozygous 1 bp deletion within exon 3 of *Hmgn1*, inducing a frameshift mutation resulting in a knockout. *Hmgn2*^−/−^ mESCs contain a homozygous 1 bp insertion within exon 4 of *Hmgn2*, inducing a frameshift mutation resulting in a knockout. *Hmgn1*^*−/−*^*Hmgn2*^*−/−*^ mESCs were generated *via* transfecting *Hmgn2*^*−/−*^ mESCs with the sgRNA-containing plasmid targeting *Hmgn1 via* the methods described above. *Hmgn1*^*−/−*^*Hmgn2*^*−/−*^ mESCs contain a homozygous 1 bp deletion within exon 3 of *Hmgn1* and a homozygous 1 bp insertion within exon 4 of *Hmgn2*, inducing a frameshift mutation within both genes, resulting in an *Hmgn1* and *Hmgn2* double knockout.

### RNA extraction

Cells were collected from 6-well plates in three biological triplicates for all downstream RNA analysis (RT–qPCR and RNA-Seq). Cells were collected and suspended in Trizol (Invitrogen; catalog no.: 15596018) and incubated for 10 min. RNA was extracted and purified using the Zymo Direct-zol RNA MiniPrep kit (Zymo; catalog no.: R2050).

### RT–qPCR

Three technical replicates were performed for each of the three biological replicates for every genotype (WT mESCs, *Hmgn1*^*−/−*^ mESCs, *Hmgn2*^*−/−*^ mESCs, and *Hmgn1*^*−/−*^*Hmgn2*^*−/−*^ mESCs) for RT–qPCR experiments. RNA was converted to complementary DNA utilizing the SuperScript IV First Strand Synthesis kit (Thermo Fisher Scientific; catalog no.: 18080-093). RT–qPCR was performed using PowerUp SYBR Green Master Mix (Applied Biosciences; catalog no.: A25742). Data are represented as the mean average FC ± standard deviation of the nine total replicates per genotype. Significance between WT and knockout cell lines was determined for each primer set using a two-tailed unpaired *t* test with asterisks indicating significance as depicted in figure legends. Significance values from paired two-tailed *t* tests are included in Table S5. Primers used for RT–qPCR experiments are included in Table S7.

### RNA-Seq and analysis

Three biological replicates were used for each genotype (WT mESCs, *Hmgn1*^*−/−*^ mESCs, *Hmgn2*^*−/−*^ mESCs, and *Hmgn1*^*−/−*^*Hmgn2*^*−/−*^ mESCs) for RNA-Seq experiments. Libraries were prepared with poly-A transcript enrichment by Novogene and sequenced on a NovaSeq 6000 instrument with 150 bp paired-end reads.

Reads were aligned to the mm10 genome using STAR (version 2.6.0). Expressed genes were defined as genes with an average reads per kilobase of transcript per million mapped read counts value ≥22, whereas nonexpressed genes were defined as genes with an average reads per kilobase of transcript per million mapped read count value <22 as defined by EMBL Expression Atlas. DEGs were identified with DESeq2 (*p*-adjusted  < 0.01, log2 FC ≥|1|) (53). Overlaps of DEGs were identified using dplyr (54). Correlation plots were created using GraphPad Prism (version 10.4.2) by GraphPad Software, LLC and analyzed with Pearson’s correlation. Heatmaps displaying log2 FCs in gene expression were generated using the pheatmap package (Research Resource Identifier: SCR_016418). Bar graphs representing the log2 FC in expression of High Mobility Group and cell identity genes were created using Microsoft Excel, with statistical significance determined by DESeq2. Principal component analysis plots were generated using the plotPCA function in DESeq2. Gene Ontology analysis was performed using Panther (released June 17, 2024), and a bubble plot was generated in R using ggplot2 (55).

### Western blotting

Cells grown for Western blotting were collected *via* 5 min of incubation with trypsin (Gibco; catalog no.: 12604-013) at 37 °C, followed by quenching with 3x volume of cell growth media or *via* scraping and collecting with PBS (Thermo Fisher Scientific; catalog no.: 10010023).

Whole cell extracts were collected by resuspending cell pellets in radioimmunoprecipitation assay buffer (50 μl per 1 million cells, 50 mM Tris–HCl, pH 8.0, 150 mM NaCl, 1% NP-40, 0.5% sodium deoxycholate, 0.1% SDS, 1X protease inhibitor cocktail (PIC) (Sigma–Aldrich; catalog no.: 11697498001), 1x PMSF, 250 U/ml Pierce Universal Nuclease (Thermo Fisher Scientific; catalog no.: 88700). Sodium butyrate (10 mM final concentration) (BioVision; catalog no.: 1609-1000) was added to the radioimmunoprecipitation assay buffer when performing extractions used to blot for histone PTMs. Resuspended cells were incubated at room temperature for 15 min (with resuspension *via* pipetting or light vortexing every ∼5 min) followed by a 15 min incubation on ice (with resuspension *via* pipetting or light vortexing every ∼5 min). Samples were centrifuged at 4 °C at maximum speed for 10 min. The supernatant was collected and used for downstream assays or frozen at −80 °C.

Nuclear extracts were collected by resuspending cell pellets in lysis buffer (10 mM Hepes [pH 7.5]; Thermo Fisher Scientific, catalog no.: 15630080, 10 mM KCl, 0.1 mM EDTA, and 0.1 mM EGTA) containing 1X PIC (Sigma–Aldrich; catalog no.: 11697498001) and were then rocked at 4 °C for 15 min. After rocking, 1 ml of 10% NP-40 was added to the samples, and they were then immediately vortexed and pelleted at 1350*g* for 5 min at 4 °C. The supernatant (cytoplasmic fraction) was collected, and the pellet (nuclear fraction) was resuspended in 1 ml of cold buffer TEN250/0.1 (50 mM Tris–HCl [pH 7.5], 250 mM NaCl, 5 mM EDTA, and 0.1 mM NP-40) containing 1X PIC and was rotated for a minimum of 30 min at 4 °C. Samples were then centrifuged at maximum speed for 10 min at 4 °C. The supernatant (nuclear extract) was collected and used for downstream assays or stored at 80 °C for long-term storage. Protein levels were quantified using the Qubit Protein BR assay kit (Thermo Fisher Scientific; catalog no.: A50668).

Samples were run on 4% to 20% Tris–glycine gels (Bio-Rad; catalog no.: 4568094) and transferred to polyvinylidene difluoride membranes (VWR; catalog no.: BSP0161). Membranes were blocked for 45 min with 5% blocking grade buffer (Bio-Rad; catalog no.: 1706404) and rocked overnight at 4 °C with primary antibody. Secondary antibody incubations were for 1 h, rocking at room temperature. All antibody washes were with 1X Tris-buffered saline with Tween-20 for 10 min and repeated for a total of three washes at room temperature. Membranes were imaged using either Thermo SuperSignal West Pico PLUS (catalog no.: 34577) or Thermo SuperSignal West Femto (catalog no.: 34094) chemiluminescent substrates with an Amersham Imager 600 (GE Healthcare). The open-source software Fiji was used to quantify blots (56). Normalization of various blots is described in the respective figure legends.

The following validated primary antibodies were used for Western blotting: SMC1 (Bethyl; catalog no.: A300-055A), SMC3 (Abcam; catalog no.: ab9263), RAD21 (Bethyl; catalog no.: A300-080A), CTCF (Active Motif; catalog no.: 31917004), Histone H3 (Abcam; catalog no.: ab1791), Actin (Abcam; catalog no.: ab190476), HMGN2 (Cell Signaling; catalog no.: 9437S), H3K27ac (Abcam; catalog no.: ab4729), H3K4me3 (Abcam; catalog no.: ab8580), H3K9ac (Millipore; catalog no.: 07-352), H3K18ac (Millipore; catalog no.: 07-354), and H3K23ac (Abcam; catalog no.: ab177275).

The following secondary antibodies were used for Western blotting: Donkey, Anti-Rabbit immunoglobulin G (horseradish peroxidase conjugated; GE Healthcare, catalog no.: NA9341ml) and Goat, Anti-Mouse immunoglobulin G (horseradish peroxidase conjugated; Invitrogen, catalog no.: A16072).

Protein signal was quantified using ImageStudioLite (LI-COR; version 5.2.5) for plots in Figure S6. Data for each histone PTM were normalized to its respective H3 lane. H3K18ac, H3K23ac, and H3K27ac data in Figure S6 were visualized in dot/line plots across the A-485 (or DMSO) washout recovery time points. Data were normalized by normalizing the signal of each histone PTM, normalized to its respective H3 lane, and then dividing by the signal in the DMSO control lane.

### ChIP-Seq

Two biological replicates were used for each genotype (WT mESCs, *Hmgn1*^*−/−*^ mESCs, *Hmgn2*^*−/−*^ mESCs, and *Hmgn1*^*−/−*^*Hmgn2*^*−/−*^ mESCs) for ChIP-Seq experiments. Cells were collected *via* trypsin (Gibco; catalog no.: 12604-013) and counted (10 million cells per immunoprecipitation [IP] condition) prior to crosslinking. Cells were crosslinked with 1% formaldehyde (Thermo Fisher Scientific; catalog no.: 28906) in PBS for 5 min, then quenched with 2.5 M glycine, and flash frozen and kept at −80 °C until use.

Crosslinked cells were resuspended in cold lysis buffer 1 (50 mM Hepes–KOH [pH 7.5], 140 mM NaCl, 1 mM EDTA, 10% glycerol, 0.5% NP-40, 1X PIC [Sigma–Aldrich; catalog no.: 11697498001], and 0.25% Triton X-100) and rotated at 4 °C for 10 min followed by centrifugation at 1350*g* for 5 min. Nuclei were lysed in room temperature lysis buffer 2 (10 mM Tris–HCl [pH 8], 200 mM NaCl, 1 mM EDTA, 1x PIC, and 0.5 mM EGTA) and rotated at room temperature for 10 min, followed by centrifugation at 1350*g* for 5 min. Chromatin pellets were then washed with cold shearing buffer (10 mM Tris [pH 7.6], 1 mM EDTA, 0.1% SDS, and 1x PIC) and then spun at 1350*g* for 5 min. RAD21 and CTCF ChIPs were resuspended in 1 ml shearing buffer with a 5% human embryonic kidney 293T chromatin spike-in (following the same procedure), and chromatin was sonicated using a Covaris E220 in milliTUBEs (Covaris; catalog no.: 520130) (with the following settings: duty factor 5, PIP/W 140, and 200 cycles per burst for 12 min) to achieve chromatin fragments of 200 to 1000 base pairs. Insoluble material was pelleted and removed by centrifuging sonicated samples for 10 min at 15,000 rpm at 4 °C.

ChIPs were performed using the antibodies referred to in Table S1. The RAD21 antibody was incubated with 50 μl of Protein A Dynabeads (Thermo Fisher Scientific; catalog no.: 10002D), using 1 μg of antibody. The CTCF antibody was incubated with 30 μl of Protein A Dynabeads (Thermo Fisher Scientific; catalog no.: 10002D) using 10 μg of antibody. Antibody–bead mixtures were incubated for 6 to 8 h with end-over-end rotation prior to the IP. After incubation, beads were washed three times with PBS supplemented with 1X PIC (Sigma–Aldrich; catalog no.: 11697498001) to remove the unbound antibody prior to the addition of chromatin.

The sonicated chromatin in shearing buffer was supplemented with NaCl and Triton X-100 to a final concentration of 150 mM NaCl and 1% Triton X-100. Sonicated chromatin from 10 million cells was added to the antibody-conjugated beads and incubated overnight at 4 °C while rotating.

Beads were then washed sequentially with ChIP buffer (20 mM Tris [pH 7.5], 2 mM EDTA, 0.1% SDS, 150 mM NaCl, and 1% Triton X-100), wash buffer 1 (20 mM Tris–HCl [pH 8], 500 mM NaCl, 2 mM, EDTA, 0.1% SDS, and 1% Triton X-100), wash buffer 2 (10 mM Tris–HCl [pH 8], 250 mM LiCl, 1 mM EDTA, and 1% NP-40), and wash buffer 3 (10 mM Tris [pH 8], 1 mM EDTA, and 50 mM NaCl), each for 5 min rotating at 4 °C.

Chromatin was then eluted from the beads by adding 100 μl IP elution buffer (50 mM Tris [pH 8], 10 mM EDTA, and 1% SDS) with incubating at 65 °C for 1 h, vortexing every 5 to 10 min. Eluted chromatin was then incubated with 5 μl 20 mg/ml Proteinase K (Thermo Fisher; catalog no.: 25-530-049) at 65 °C overnight to reverse crosslinking. DNA was then purified using a ChIP DNA Clean and Concentrate kit (Zymo; D5205) and eluted in 30 μl Zymo elution buffer.

Libraries were prepped using the Kapa HyperPrep kit following the manufacturer’s instructions (Roche/Kapa; KK8502). Sequencing was performed on a NovaSeq 6000 SP, collecting 50 bp paired-end reads.

### ChIP-Seq analysis

ChIP-Seq was analyzed utilizing previously published and custom scripts found on GitHub (https://github.com/dowenlab). Raw fastq files of biological replicates for each sample were concatenated prior to alignment. Merged replicates were aligned to a merged genome containing both mouse (mm10) and human (hg38) genome assemblies using bowtie (v1.3.1) (parameters -v 2 -p 24 -S -m 1 -best -strata) (57). Mouse chromosomes were denoted with an Mchr prefix to distinguish them from human chromosomes. Duplicate sequences were removed using samtools (v1.17) markdup (-r -s) (58). A bam file containing only mouse reads was created using samtools view and converted to bed format with bedtools (v2.25.0) bamtobed (59). Reads were extended by 200 bp on both sides and then used to call peaks using MACS (version 2.1.2) with a false discovery rate of 1% (macs2 callpeak -f BED -g mm -q 0.01) (60). Peak summits were expanded by 50 bp on both sides. Peaks that overlap regions with repetitive sequences, as defined in the ENCODE mm10 blacklist, were removed using bedtools intersect (-v) (61).

To establish a high-confidence peak list by removing false-positive peaks, appropriate samples were filtered for peaks based on *q* values. HMGN1 peaks were filtered for peaks with *q* values >15, HMGN2 peaks were filtered for peaks with *q* values >5, and H3K4me3 peaks were filtered for peaks with *q* values >2. H3K27ac and H2A.Z peaks were not filtered. For all plots and analyses in Figure 1, RAD21 and CTCF ChIP-Seq data are a merge of the following datasets: RAD21: GSM8768208, GSM8768209, GSM8768210, GSM8768211, and GSM8768212; CTCF: GSM8768213, GSM8768214, GSM8768215, GSM8768216, and GSM8768217. These merged CTCF peaks were filtered with *q* values >8, and the merged RAD21 peaks were not filtered. For all plots and analyses in Figure 3, WT RAD21 and CTCF mESC ChIP-Seq data include the following datasets: RAD21: GSM8768211, GSM8768212; CTCF: GSM8768216, GSM8768217. The merged CTCF peaks in WT mESCs and *Hmgn1*^*−/−*^*Hmgn2*^*−/−*^ mESCs were not filtered, and the merged RAD21 peaks in WT mESCs and *Hmgn1*^*−/−*^*Hmgn2*^*−/−*^ mESCs were filtered with *q* values >8. The resulting peak files were extended 200 bp on both sides and used for downstream analyses. Sequencing statistics and sample information can be found in Table S2.

For datasets that included a spike-in (RAD21, CTCF, and H3K27ac), a normalization factor was calculated by counting mouse and human nonduplicate reads with samtools idxstats and awk. Each ChIP-Seq dataset was then designated a normalization factor (normFactor) using the formula 5/((h/m)∗100), where h is the number of human reads in millions and m is the number of mouse reads in millions. For datasets that did not include a spike-in (HMGN1, HMGN2, H3K4me3, and H2A.Z), no scaling factor was applied. The bed file containing mouse reads was converted to a bedgraph file using bedtools genomecov (-bga -scale normFactor) and then converted to a bigwig file with bedGraphToBigWig from ucsctools (version 320) (62). Z-score normalization of bigwig files was performed as indicated using a custom R script from Dr Spencer Nystrom of Dr Daniel McKay’s laboratory.

Signal tracks for ChIP-Seq data were visualized using IGV 2.4.10 desktop browser (63). Genes bound by HMGN1 and/or HMGN2 were determined in R using bedtoolsr with a union peak list of HMGN1 and HMGN2 peaks in WT mESCs (64). Correlation heatmaps were generated using deeptools (version 3.2.0) multiBigwigSummary, followed by plotCorrelation using Pearson's correlation (65). Peak overlaps were determined using bedtools intersect (59). UpSet plots were generated in R (version 4.1.0) using bedtoolsr and UpSetR using HMGN1 peaks and HMGN2 peaks in WT mESCs overlapping, RAD21 peaks, CTCF peaks, H3K27ac peaks, H3K4me3 peaks, H2A.Z peaks, and UCSC TSSs (66). “Other” sites are those remaining after taking the peak list of interest (HMGN1 or HMGN2 peaks) and removing sites that overlapped with RAD21 sites, CTCF sites, H3K27ac sites, H3K4me3 sites, H2A.Z sites, and UCSC TSSs using bedtools intersect (−v). Average signal plots were generated using deeptools (version 3.0.1) computeMatrix (reference point), followed by plotProfile. Heatmaps were generated using z-score normalized bigwig files with deeptools (version 2.4.1) computeMatrix (reference point) followed by deeptools plotHeatmap. Active enhancers were defined as sites with H3K27ac in WT mESCs. Active promoters were defined as UCSC TSSs that overlap H3K4me3 peaks. Insulator sites were defined as RAD21 and CTCF sites that did not overlap active enhancers and active promoters. Fingerprint plots were generated using deeptools (version 3.0.1) plotFingerprint (–skipZeros). Differentially bound sites and corresponding MA plots were generated using DiffBind in R (version 4.1.0) (67).

### Expression and purification of GST-tagged HMGN proteins

Plasmids containing the N-terminal tagged GST protein of interest (HMGN1, HMGN2, HMGN1ΔC, or HMGN2ΔC) were transformed into chemically competent BL21.DE3(pLysS) *E*. *coli* cells, then plated onto LB-agar plates containing 50 μg/ml carbenicillin and incubated overnight at 37 °C. Five to 10 colonies were inoculated into 250 ml LB media containing 50 μg/ml carbenicillin and incubated at 37 °C until absorbance was achieved (absorbance at 600 nm = 0.5–0.8). After absorbance was achieved, cultures were induced with 1 mM IPTG (RPI; catalog no.: 156000) and incubated at 18 °C for 18 to 20 h. Following induction, cultures were harvested by centrifugation, and cell pellets were flash frozen in liquid nitrogen and stored at −80 °C until use.

Cell pellets were resuspended in 50 ml of lysis buffer (PBS, 300 mM NaCl, 1 mM DTT, 1 mM PMSF, 1x PIC, 1 mg/ml lysozyme [Sigma; catalog no.: L6876], and 250 U of Pierce Universal Nuclease [Thermo Fisher Scientific; catalog no.: 88701], and incubated at 37 °C for 10 min). Cells were then lysed by probe sonication (4–5 × 14 s, 15% power), and the lysate was clarified by centrifugation. Clarified lysate was applied to a glutathione-agarose bead column. The column was washed with 20 column volumes of wash buffer (wash buffer: 50 mM Hepes [pH 7.5] [Thermo Fisher Scientific; catalog no.: 15630080], 1 mM PMSF, 500 mM NaCl, 1x PIC). Bound GST-tagged proteins were then eluted in five low-GSH fractions (low GSH buffer: 50 mM Hepes [pH 7.5] [Thermo Fisher Scientific; catalog no.: 15630080], 1 mM PMSF, 500 mM NaCl, 1x PIC, 1 mM GSH [Thermo Fisher Scientific; catalog no.: 78259], and five high-GSH fractions [high GSH buffer: 50 mM Hepes [pH 7.5], 1 mM PMSF, 500 mM NaCl, 1x PIC, and 10 mM GSH]). Fractions containing pure GST-tagged proteins were pooled and concentrated by centrifugation filtration (Thermo Fisher Scientific; catalog no.: 88516; using appropriate molecular weight cutoff, either 10 kDa or 30 kDa) and then buffer exchanged into storage buffer (50 mM Hepes, pH 7.5, 500 mM NaCl, 1 mM DTT, 0.5 mM PMSF, and 20% glycerol). Concentrated pools were aliquoted and quantified by Coomassie-stained SDS-PAGE–based densitometry with known bovine serum albumin (BSA) standards and stored at −80 °C until use.

### Nucleosome binding assays

Individual biotinylated mononucleosomes obtained from Epicypher (Epicypher 16-0006, 16-0029, 16-0030, 16-0014, 16-0316, 16-0314, 16-0343, 16-0372, 16-0364, 16-0365, 16-0016, 16-0027, 16-0315, 16-0317, and 16-0320) were conjugated to spectrally distinct streptavidin-coated carboxylated polystyrene MagPlex beads (Diasorin) and pooled into a panel to contain 10 million beads/ml of each nucleosome-bead conjugate. A final concentration of 20,000 beads/ml of the nucleosome-bead conjugate panel and titrations of 0 to 5 nM GST-tagged protein (HMGN1, HMGN2, HMGN1ΔC, or HMGN2ΔC) diluted in nucleosome wash buffer (50 mM NaCl, 25 mM Hepes [pH 7.5] [Thermo Fisher Scientific; catalog no.: 15630080], 1 mM DTT, 0.5% BSA, and 0.1% Tween-20) was added to a flat bottom 96-well plate and incubated at room temperature for 2 h on a rocker at 650 rpm. After incubation, beads were magnetized on a 96-well plate magnet and washed with nucleosome wash buffer twice. Anti-GST primary antibody (Thermo Fisher Scientific; catalog no.: A190-122A) at 1:1000 dilution was added to the wells. Antihistone antibody (Millipore; catalog no.: MAB3422) at 1:200 dilution or anti-DNA antibody (Millipore; catalog no.: MAB1293) at 1:200 dilution was added to a control well to measure intact nucleosomes. Primary antibody was added at a final volume of 100 μl per well and incubated at room temperature for 45 min on a rocker at 650 rpm. Wells were magnetized and washed twice after primary antibody incubation. Secondary antibody conjugated to phycoerythrin (Thermo; catalog no.: P-2771MP or P-852) in 100 μl per well was added to each well and incubated at room temperature for 45 min on a rocker at 650 rpm. Wells were magnetized and washed thrice and read on a Luminex xMAP INTELLIFLEX System (Diasorin).

Raw fluorescent data were visualized in a dot/line plot across the titrations of protein for each nucleosome-bead conjugate as well as visualized as normalized data. Background signal captured by 50 mM BSA-bead, 100 mM BSA-bead, and 200 mM BSA-bead conjugates, as well as wells containing 0 mM HMGN protein, were subtracted from raw values for nucleosome-bead conjugates and then normalized to the unmodified H3.1 mononucleosome-bead conjugate and plotted as a relative bar plot. Significance values from paired two-tailed *t* tests for nucleosome binding assays are found in Table S5.

### HAT assays

HAT assays were performed at 37˚C for 30 min with 500 nM purified p300 (BPS Biosciences; catalog no.: 50071), 300 nM nucleosome substrate (Epicypher 16-0009, 16-1014, or 16-1014), 100 μM acetyl-CoA (Sigma; catalog no.: A2056), and 100 nM, 300 nM, or 900 nM purified GST-HMGN protein (HMGN1, HMGN2, HMGN1ΔC, or HMGN2ΔC) in a reaction volume of 10 μl (in HAT buffer: 50 nM Tris–HCl [pH 8.0], 10% glycerol, 1 mM DTT, 0.1 mM EDTA, 1 mM PMSF, and 10 mM sodium butyrate). Optimal p300 enzyme concentration for endpoint analysis was identified through an enzyme titration (0–500 nM) against 300 nM recombinant human H3.1 mononucleosome (Epicypher, 16-0006) at 37 °C for 30 min. Nucleosome substrate, purified HMGN protein, and HAT buffer were first incubated for 30 min on ice prior to the addition of purified p300 to allow for HMGN–nucleosome complexes to form. After 30 min of incubation at 37 °C, the reaction was stopped by adding 4x Laemmli buffer (Bio-Rad; catalog no.: 1610747) and then boiled for 10 min at 95 °C. Samples were run on a 4% to 20% SDS-polyacrylamide gel (Bio-Rad; catalog no.: 4568094 or 4568093). To access histone H3 acetylation, commercially available antibodies against H3K18ac (Millipore; catalog no.: 07-354), H3K23ac (Abcam; catalog no.: ab177275), and H3K27ac (Abcam; catalog no.: ab4729) were utilized. To assess p300 and HMGN protein, anti-GST antibody (Pocono, gifted from the Strahl Lab at UNC Chapel Hill) and anti-HMGN2 antibody (Cell Signaling; catalog no.: 9437S) were utilized, and to assess total H3, anti-H3 antibody (Abcam; catalog no.: ab1791) was utilized.

H3K18ac, H3K23ac, and H3K27ac data were visualized in dot/line plots across the titration series of HMGN protein to canonical nucleosomes. Data were normalized by calculating the signal of each acetylation mark in each titration of HMGN protein, divided by the signal in the “no HMGN” lane, and then normalized to relative H3. GraphPad Prism was used to generate dot/line plots.

### Epiproteomic histone modification analysis by mass spectrometry

WT and *Hmgn1*^*−/−*^*Hmgn2*^*−/−*^ mESCs were cultured as described above and collected in three biological replicates, each consisting of 5 million cells. Histone extraction, derivatization, and trypsin digestion were adapted from previous works and described elsewhere (68, 69). Briefly, cells were lysed for 30 min on ice with nuclear isolation buffer (NIB) to isolate intact nuclei. The NIB composition was 15 mM Tris–HCl (pH 7.5), 60 mM KCl, 15 mM NaCl, 5 mM MgCl_2_, 1 mM CaCl_2_, 250 mM sucrose, and 0.3% NP-40. DTT (1 mM), 1:100 Halt protease inhibitor, and 10 mM sodium butyrate were added immediately prior to use. Nuclei were pelleted at 600*g* for 5 min at 4 °C and then washed twice with NIB without NP-40. Histones were extracted with five volumes of 0.2 M sulfuric acid for 60 min at room temperature. Following centrifugation at 4000*g* for 5 min, trichloroacetic acid was added to the supernatant to a final concentration of 20% (v/v). Precipitated histones were pelleted at 10,000*g* for 5 min, washed once with acetone containing 0.1% HCl, then twice with only acetone. Histones were air dried and then resuspended in 10 μl of 100 mM ammonium bicarbonate for derivatization. Samples were propionylated with 30 μl of freshly prepared 1:3 (v/v) propionic anhydride to isopropanol and adjusted to pH 8 with ammonium hydroxide. Samples were derivatized for 60 min at 50 °C, dried by vacuum centrifugation, and then digested overnight with 500 ng of trypsin at 37 °C. Peptides were dried by vacuum centrifugation and then underwent an additional round of propionylation as described above. Peptides were resuspended in 50 μl of 0.1% aqueous trifluoroacetic acid in water and then transferred to glass autosampler vials prior to mass spectrometry analysis.

Multiple reaction monitoring was performed on a TSQ Altis triple quadrupole mass spectrometer coupled to an UltiMate 3000 Dionex nano HPLC system (both Thermo Fisher Scientific). Peptides were loaded onto a C18 trap column (75 μm × 2 cm, Acclaim PepMap 100) and then separated using a PicoChip analytical column (75 μm × 10 cm, ProntoSIL C18-AQ, 3 μm, 200 Å resin, New Objective PicoChip). Peptide separation was achieved using an increasing solvent gradient of mobile phase A (0.1% aqueous formic acid) and mobile phase B (95% acetonitrile and 0.1% aqueous formic acid) with conditions: 0% to 35% B at 300 nl/min flow rate over 45 min.

The following triple quadrupole settings were used across all analyses: electrospray voltage of 2.5 kV, argon collision gas pressure of 1.5 mTorr, Q1 peak width of 0.7 full width at half maximum, 2 s cycle time, and skimmer offset of 10 V. Modified and unmodified histone peptides monitored in the assay were selected based on previous reports (68, 70).

Mass spectrometry raw files were imported and analyzed in Skyline software with Savitzky–Golay smoothing (71). Automatic peak assignments from Skyline were manually confirmed, and peptide peak areas were used to determine the relative abundance of each histone modification. Peptides were considered detected if the signal-to-noise ratio was above 3 and quantifiable, and at least one modification state was detected in addition to the unmodified peptide. Results were further refined in GraphPad Prism to plot graphs representing the relative abundance of each histone modification. Relative abundances were determined based on the mean of three technical replicates, with error bars representing the standard deviation. Peptide modifications are abbreviated as un (unmodified), me1 (monomethyl), me2 (dimethyl), me3 (trimethyl), and ac (acetyl).

## Data availability

All data produced for this study are included in the article and supporting information. The ChIP-Seq and RNA-Seq datasets generated in this study are available in the National Center for Biotechnology Information Gene Expression Omnibus repository under accession numbers GSE291947 and GSE291949, respectively. Previously collected and reported ChIP-Seq data used in this study are summarized in Table S1 and can also be found in the National Center for Biotechnology Information Gene Expression Omnibus repository. The mass spectrometry proteomics data are available in the ProteomeXchange Consortium *via* the PRIDE partner repository with the dataset identifier PXD070369 (72).

## Supporting information

This article contains supporting information.

## Conflict of interests

EpiCypher is a commercial developer and supplier of fully defined semisynthetic nucleosomes used in this study. B. D. S. owns shares in EpiCypher and is a member of the Board of Directors. B. D. S. is an Associate Editor for this journal and was not involved in the editorial review or the decision to publish this article. All other authors declare that they have no conflicts of interest with the contents of this article.
