# Supplementary material for: HMGN1 and HMGN2 are recruited to acetylated and histone variant H2A.Z-containing nucleosomes to regulate chromatin state and transcription
Source: J Biol Chem. 2025 Nov 29;302(1):110997. doi: 10.1016/j.jbc.2025.110997 (PMC12796744; doi:10.1016/j.jbc.2025.110997)
Supplement: Supporting Information [file mmc8.pdf]

Supporting information for

HMGN1 and HMGN2 are recruited to acetylated and histone variant H2A.Z-containing nucleosomes to regulate chromatin state and transcription

Riya Gohil[1,2], Zhihan Gao[3], Rebecca A. Lewis[3], Nathaniel T. Burkholder[4], Brian D. Strahl[1,4,5], and Jill M. Dowen[1,2,3,4,5]*

[1]Curriculum in Genetics and Molecular Biology, University of North Carolina at Chapel Hill, Chapel Hill, NC 27599, USA.
[2]Integrative Program for Biological and Genome Sciences, University of North Carolina at Chapel Hill, Chapel Hill, NC 27599, USA.
[3]Department of Biology, University of North Carolina at Chapel Hill, Chapel Hill, NC 27599, USA.
[4]Department of Biochemistry & Biophysics, University of North Carolina at Chapel Hill, Chapel Hill, NC 27599, USA.
[5]Lineberger Comprehensive Cancer Center, University of North Carolina at Chapel Hill, Chapel Hill, NC 27599, USA.
*Corresponding Author

Supplementary Figures S1-S7

Supplementary Figure S1. Distribution of HMGN enrichment across the genome.

Supplementary Figure S2. Validation of HMGN single knockout genotypes and supporting RNA-seq data.

Supplementary Figure S3. Cohesin and CTCF ChIP-seq enrichment.

Supplementary Figure S4. Analysis of HMGN binding to nucleosomes.

Supplementary Figure S5. Histone acetyltransferase assay replicates.

Supplementary Figure S6. HMGN1 and HMGN2 do not alter the *in vivo* recovery kinetics of p300-mediated histone acetylation.

Supplementary Figure S7. Quantification of histone H3 modification states in *Hmgn1*$^{-/-}$ *Hmgn2*$^{-/-}$ mESCs by epiproteomic mass spectrometry.

Supplementary Tables S1-S7 (provided in separate Excel file)

Table S1. Accession numbers.

Table S2. QC information.

Table S3. Differentially expressed genes.

Table S4. Gene ontology analysis of differentially expressed genes.

Table S5. Significance values of statistical analyses.

Table S6. Modification states of histone tail residues.

Table S7. Oligos.

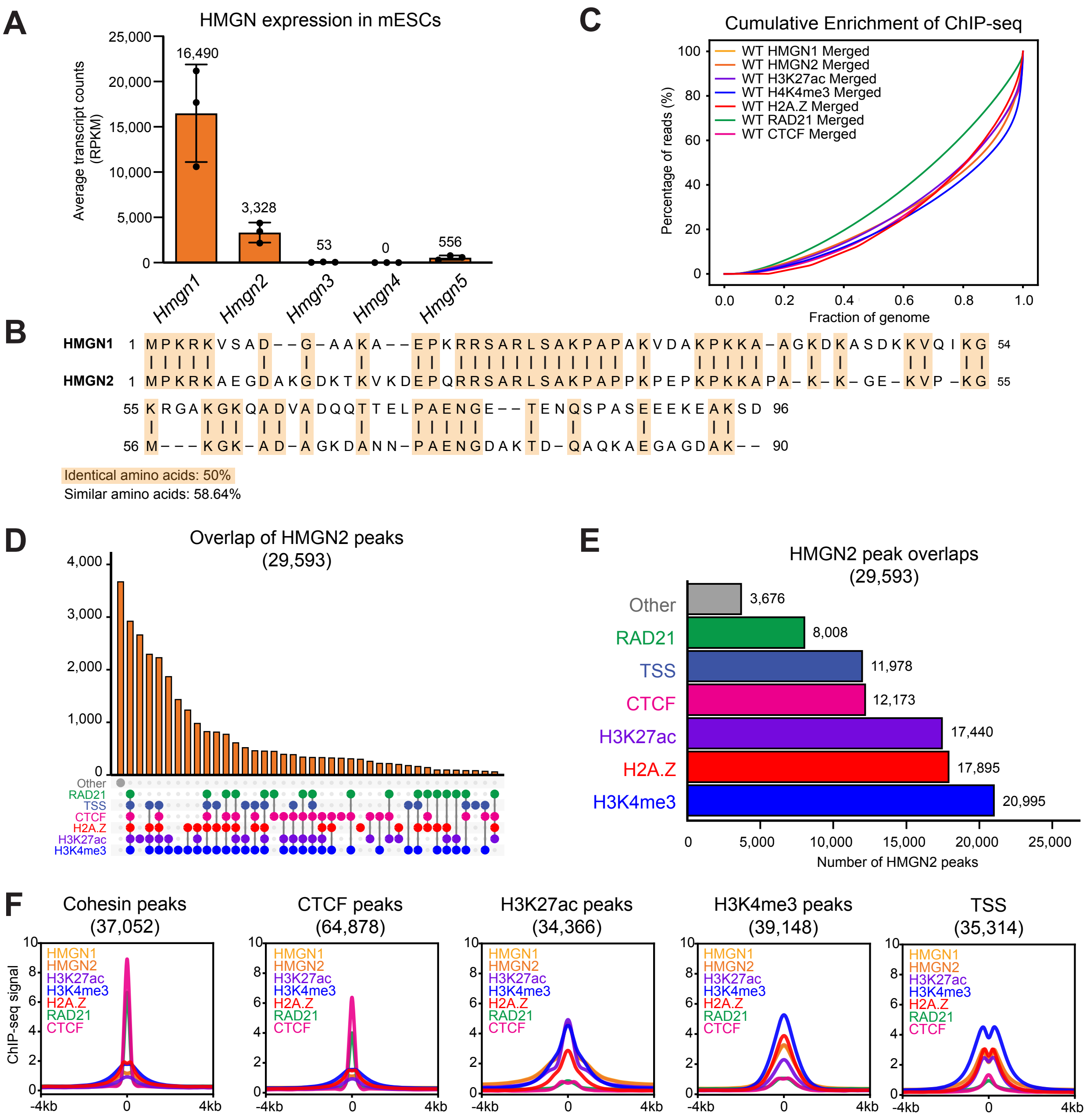

Supplementary Figure S1: Distribution of HMGN enrichment across the genome.

A) Bar graph of average transcript counts (RPKM) of *Hmgn1* mRNA, *Hmgn2* mRNA, *Hmgn3* mRNA, *Hmgn4* mRNA, and *Hmgn5* mRNA in the WT mESCs RNA-sequencing data. Error bars represent the standard deviation calculated from three biological replicates.

B) Amino acid sequence of HMGN1 and HMGN2 proteins, protein alignment, and similarity between the two proteins. Alignment was generated using VectorBuilder Sequence Alignment tool using NCBI Reference Sequence NP_032277.3 for HMGN1 amino acid sequence and NCBI Reference Sequence Accession AAI03793.1 for HMGN2 amino acid sequence.

C) Fingerprint plot showing cumulative enrichment of reads per fraction of the genome for merged replicates of HMGN1, HMGN2, H3K27ac, H3K4me3, H2A.Z, RAD21, and CTCF ChIP-seq data in WT mESCs.
D) UpSet plot of HMGN2 ChIP-seq peaks in WT mESCs displaying intersection of sets of peaks at H3K27ac, H3K4me3, transcription start sites (TSS), H2A.Z, RAD21, CTCF, and other sites.
E) Bar graph of number of HMGN2 peaks that overlap with H3K4me3, H2A.Z, H3K27ac, CTCF, transcription start sites (TSS), RAD21, and other peaks in WT mESCs.
F) Average signal plots of HMGN1, HMGN2, H3K27ac, H3K4me3, H2A.Z, RAD21, and CTCF ChIP-seq signal at cohesin peaks, CTCF peaks, H3K27ac peaks, H3K4me3 peaks, and transcription start sites (TSS).

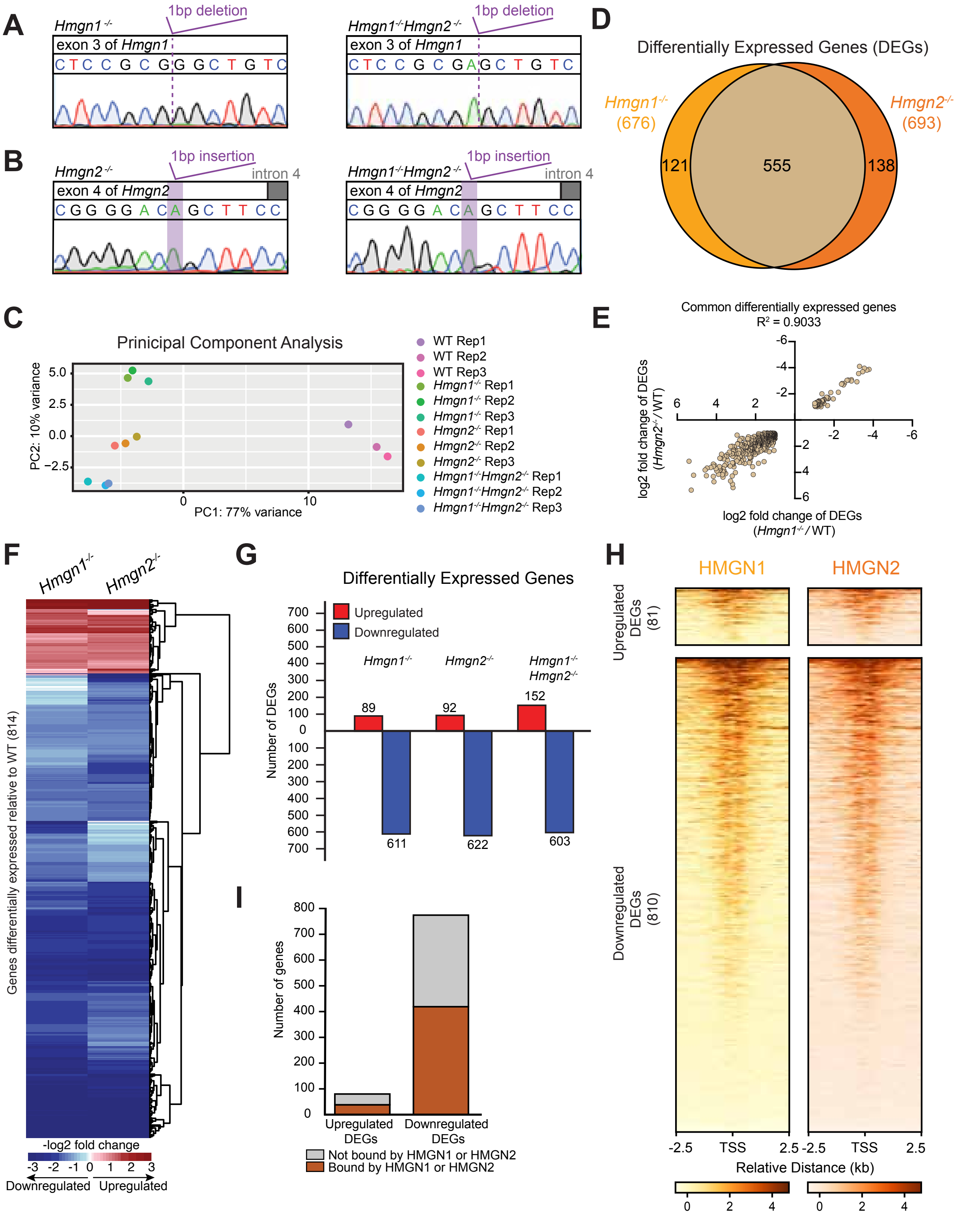

Supplementary Figure S2: Validation of HMGN single knockout genotypes and supporting RNA-seq data.

A) Sequencing chromatograms for *Hmgn1*$^{-/-}$ mESCs and *Hmgn1*$^{-/-}$*Hmgn2*$^{-/-}$ mESCs at the *Hmgn1* locus at exon 3. Purple dashed lines indicate edits.
B) Sequencing chromatograms for *Hmgn2*$^{-/-}$ mESCs and *Hmgn1*$^{-/-}$*Hmgn2*$^{-/-}$ mESCs at the *Hmgn2* locus at exon 4. Purple dashed lines indicate edits.
C) Principal Component Analysis (PCA) of RNA-seq replicates in WT mESCs, *Hmgn1*$^{-/-}$ mESCs, *Hmgn2*$^{-/-}$ mESCs, and *Hmgn1*$^{-/-}$*Hmgn2*$^{-/-}$ mESCs.
D) Overlap of differentially expressed genes (DEGs) in *Hmgn1*$^{-/-}$ mESCs and *Hmgn2*$^{-/-}$ mESCs relative to WT mESCs.
E) Correlation plot of log2 fold change in expression of Common DEGs (555) in *Hmgn1*$^{-/-}$ mESCs and *Hmgn2*$^{-/-}$ mESCs. Axes cropped, removing one outlier.
F) Clustered heatmap of -log2 fold change in expression for all DEGs in *Hmgn1*$^{-/-}$ mESCs and *Hmgn2*$^{-/-}$ mESCs.
G) Bar graph of number of DEGs in *Hmgn1*$^{-/-}$ mESCs, *Hmgn2*$^{-/-}$ mESCs, and *Hmgn1*$^{-/-}$*Hmgn2*$^{-/-}$ mESCs relative to WT mESCs.
H) ChIP-seq signal of HMGN1 and HMGN2 in WT mESCs shown at the promoters of up and downregulated DEGs in *Hmgn1*$^{-/-}$ mESCs, *Hmgn2*$^{-/-}$ mESCs, or *Hmgn1*$^{-/-}$*Hmgn2*$^{-/-}$ mESCs.
I) Bar graph of number of differentially expressed genes in the *Hmgn1*$^{-/-}$*Hmgn2*$^{-/-}$ mESCs bound and not bound by HMGN1 and/or HMGN2.

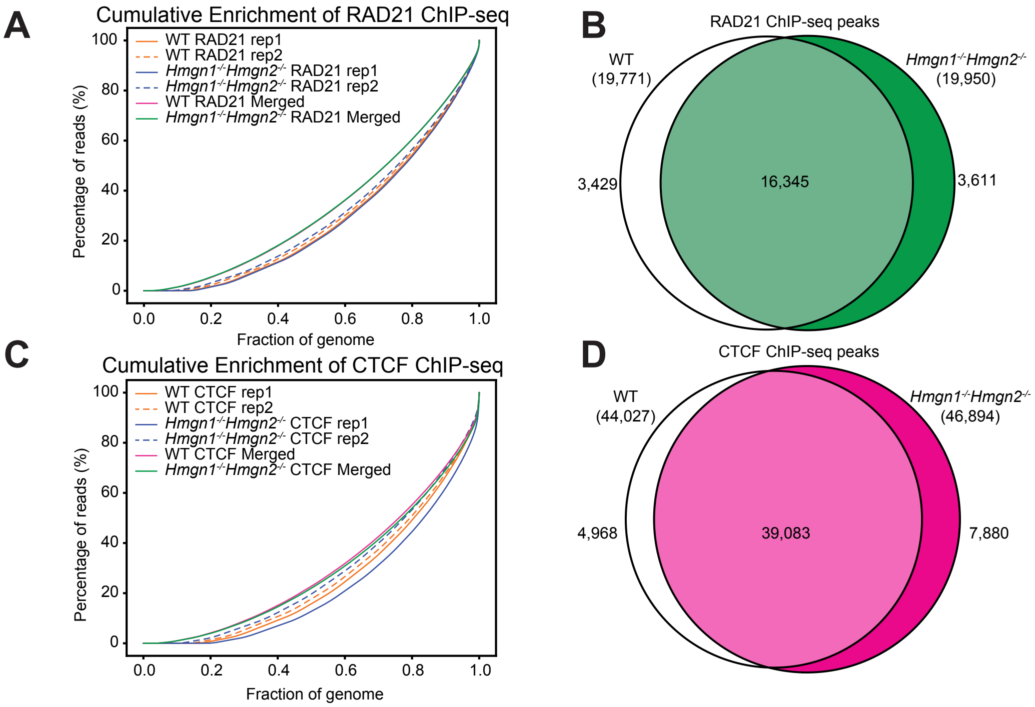

Supplementary Figure S3: Cohesin and CTCF ChIP-seq enrichment.

A) Fingerprint plot showing cumulative enrichment of reads per fraction of the genome for each biological replicate and merged replicates of RAD21 ChIP-seq data in WT mESCs and *Hmgn1*$^{-/-}$*Hmgn2*$^{-/-}$ mESCs.
B) Overlap of RAD21 ChIP-seq peaks in WT mESCs and *Hmgn1*$^{-/-}$*Hmgn2*$^{-/-}$ mESCs.
C) Fingerprint plot showing cumulative enrichment of reads per fraction of the genome for each biological replicate and merged replicates of CTCF ChIP-seq data in WT mESCs and *Hmgn1*$^{-/-}$*Hmgn2*$^{-/-}$ mESCs.
D) Overlap of CTCF ChIP-seq peaks in WT mESCs and *Hmgn1*$^{-/-}$*Hmgn2*$^{-/-}$ mESCs.

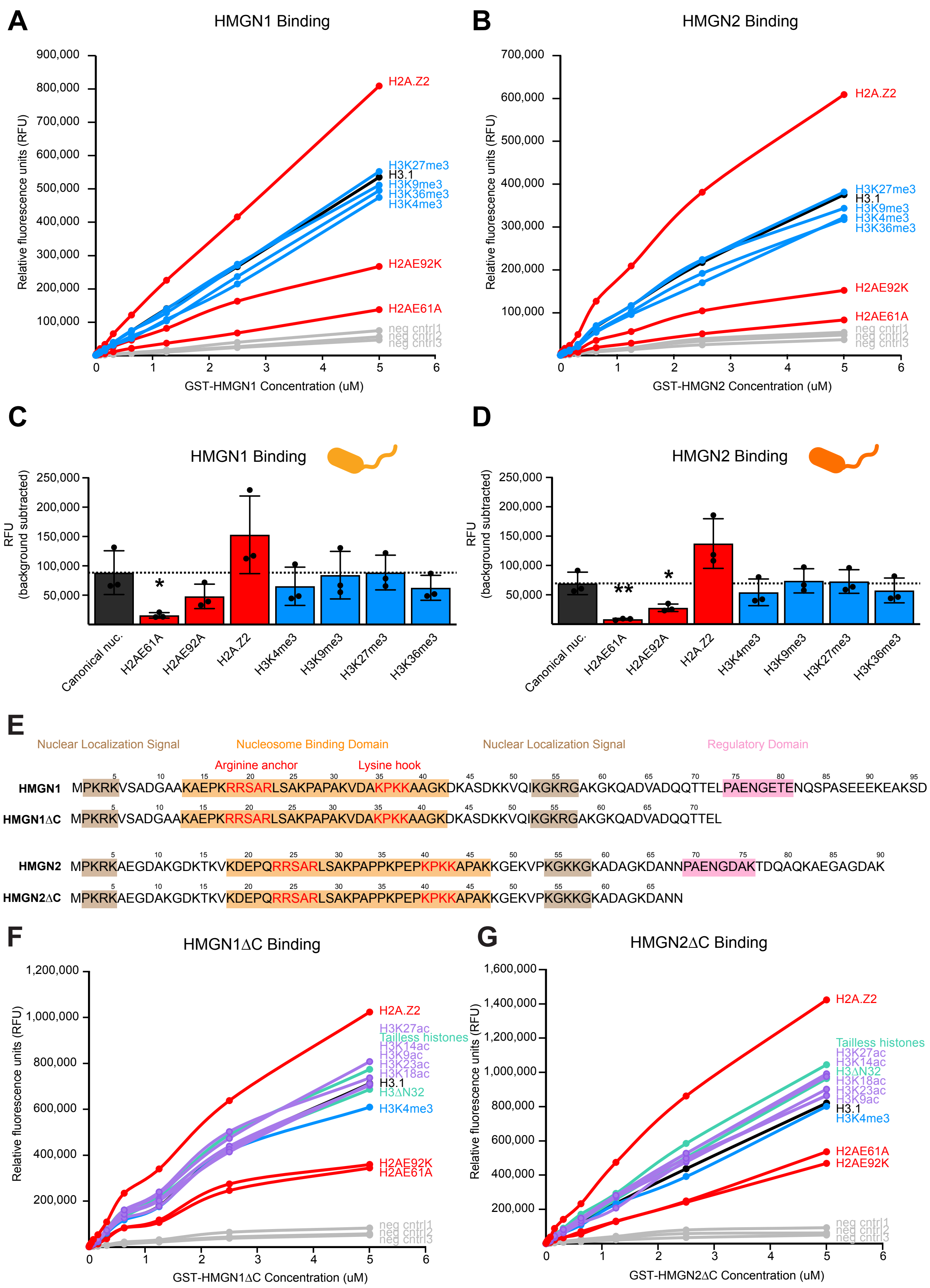
A
HMGN1 Binding
Relative fluorescence units (RFU)
GST-HMGN1 Concentration (uM)
H2A.Z2
H3K27me3
H3.1
H3K9me3
H3K36me3
H3K4me3
H2AE92K
H2AE61A
neg cntrl1
neg cntrl2
neg cntrl3
B
HMGN2 Binding
Relative fluorescence units (RFU)
GST-HMGN2 Concentration (uM)
H2A.Z2
H3K27me3
H3.1
H3K9me3
H3K4me3
H3K36me3
H2AE92K
H2AE61A
neg cntrl1
neg cntrl2
neg cntrl3
C
HMGN1 Binding
RFU
(background subtracted)
Canonical nuc.
H2AE61A
H2AE92A
H2A.Z2
H3K4me3
H3K9me3
H3K27me3
H3K36me3
D
HMGN2 Binding
RFU
(background subtracted)
Canonical nuc.
H2AE61A
H2AE92A
H2A.Z2
H3K4me3
H3K9me3
H3K27me3
H3K36me3
E
Nuclear Localization Signal
Nucleosome Binding Domain
Nuclear Localization Signal
Regulatory Domain
Arginine anchor
Lysine hook
HMGN1 MPKRKVSADGAAKAEPKRRSARLSAKPAPAKVDAKPKKAAGKDKASDKKVQIKGKRGAKGKQADVADQQTTELPAENGETENQSPASEEEKEAKSD
HMGN1ΔC MPKRKVSADGAAKAEPKRRSARLSAKPAPAKVDAKPKKAAGKDKASDKKVQIKGKRGAKGKQADVADQQTTEL
HMGN2 MPKRKAEGDAKGDKTKVKDEPQRRSARLSAKPAPPKPEPKPKKAPAKKGEKVPKGKKGKADAGKDANNPAENGDAKTDQAQKAEGAGDAK
HMGN2ΔC MPKRKAEGDAKGDKTKVKDEPQRRSARLSAKPAPPKPEPKPKKAPAKKGEKVPKGKKGKADAGKDANN
F
HMGN1ΔC Binding
Relative fluorescence units (RFU)
GST-HMGN1ΔC Concentration (uM)
H2A.Z2
H3K27ac
Tailless histones
H3K14ac
H3K9ac
H3K23ac
H3K18ac
H3.1
H3ΔN32
H3K4me3
H2AE92K
H2AE61A
neg cntrl1
neg cntrl2
neg cntrl3
G
HMGN2ΔC Binding
Relative fluorescence units (RFU)
GST-HMGN2ΔC Concentration (uM)
H2A.Z2
Tailless histones
H3K27ac
H3K14ac
H3ΔN32
H3K18ac
H3K23ac
H3K9ac
H3.1
H3K4me3
H2AE61A
H2AE92K
neg cntrl1
neg cntrl2
neg cntrl3

Supplementary Figure S4: Analysis of HMGN binding to nucleosomes.

A) Titration of GST-HMGN1 protein with each nucleosome-bead conjugate, expressed as relative fluorescence units before normalization.
B) Titration of GST-HMGN2 protein with each nucleosome-bead conjugate, expressed as relative fluorescence units before normalization.
C) GST-HMGN1 binding relative to canonical nucleosome with background subtracted. Background signal captured by negative control bead conjugates (Average signal of 50mM BSA-bead, 100mM BSA-bead, and 200mM BSA-bead conjugates) and wells containing 0mM GST-HMGN1 protein were subtracted from raw values for each nucleosome-bead conjugates at 0.625nM GST-HMGN1 concentration. A t-test was used to assess statistical significance with one asterisk (*) denoting a p-value less than 0.05, ** indicating a p-value less than 0.01, and *** representing a p-value less than 0.001. Error bars represent the standard deviation calculated from three technical replicates.
D) GST-HMGN2 binding relative to canonical nucleosome with background subtracted. Background signal captured by negative control bead conjugates (Average signal of 50mM BSA-bead, 100mM BSA-bead, and 200mM BSA-bead conjugates) and wells containing 0mM GST-HMGN2 protein were subtracted from raw values for each nucleosome-bead conjugates at 0.625nM GST-HMGN2 concentration. A t-test was used to assess statistical significance with one asterisk (*) denoting a p-value less than 0.05, ** indicating a p-value less than 0.01, and *** representing a p-value less than 0.001. Error bars represent the standard deviation calculated from three technical replicates.
E) Amino acid sequence of HMGN1 and HMGN2 proteins of purified GST-tagged HMGN1, GST-tagged HMGN2, GST-tagged HMGN1ΔC, and GST-tagged HMGN2ΔC proteins. Domains are highlighted.
F) Titration of GST-HMGN1ΔC protein with each nucleosome-bead conjugate, expressed as relative fluorescence units before normalization.
G) Titration of GST-HMGN2ΔC protein with each nucleosome-bead conjugate, expressed as relative fluorescence units before normalization.

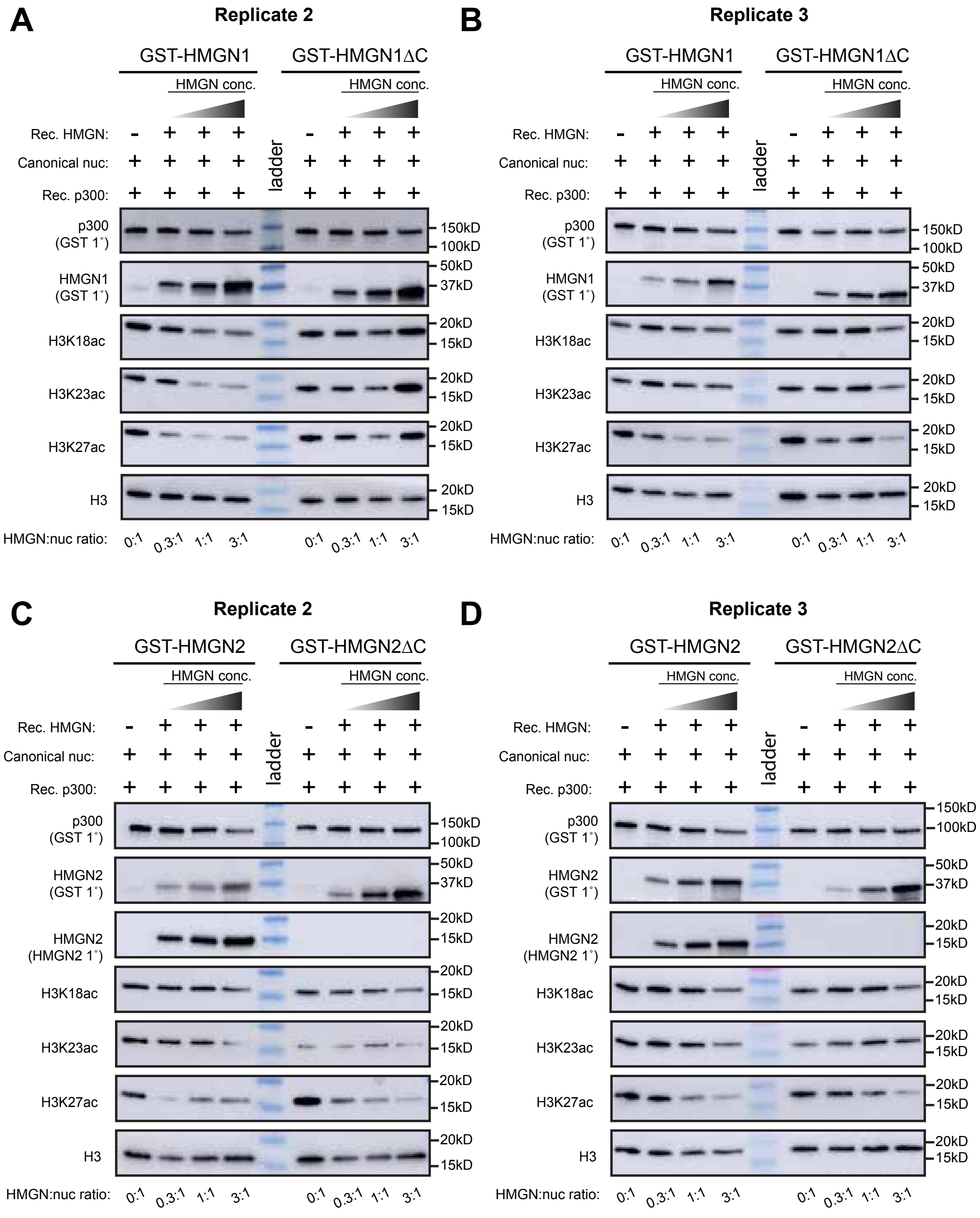

Supplementary Figure S5: Histone acetyltransferase assay replicates.

A) Replicate 2 of Western blot analysis of p300 HAT with recombinant GST-HMGN1 or GST-HMGN1ΔC protein.
B) Replicate 3 of Western blot analysis of p300 HAT with recombinant GST-HMGN1 or GST-HMGN1ΔC protein.
C) Replicate 2 of Western blot analysis of p300 HAT with recombinant GST-HMGN2 or GST-HMGN2ΔC protein.

D) Replicate 3 of Western blot analysis of p300 HAT with recombinant GST-HMGN2 or GST-HMGN2ΔC protein.

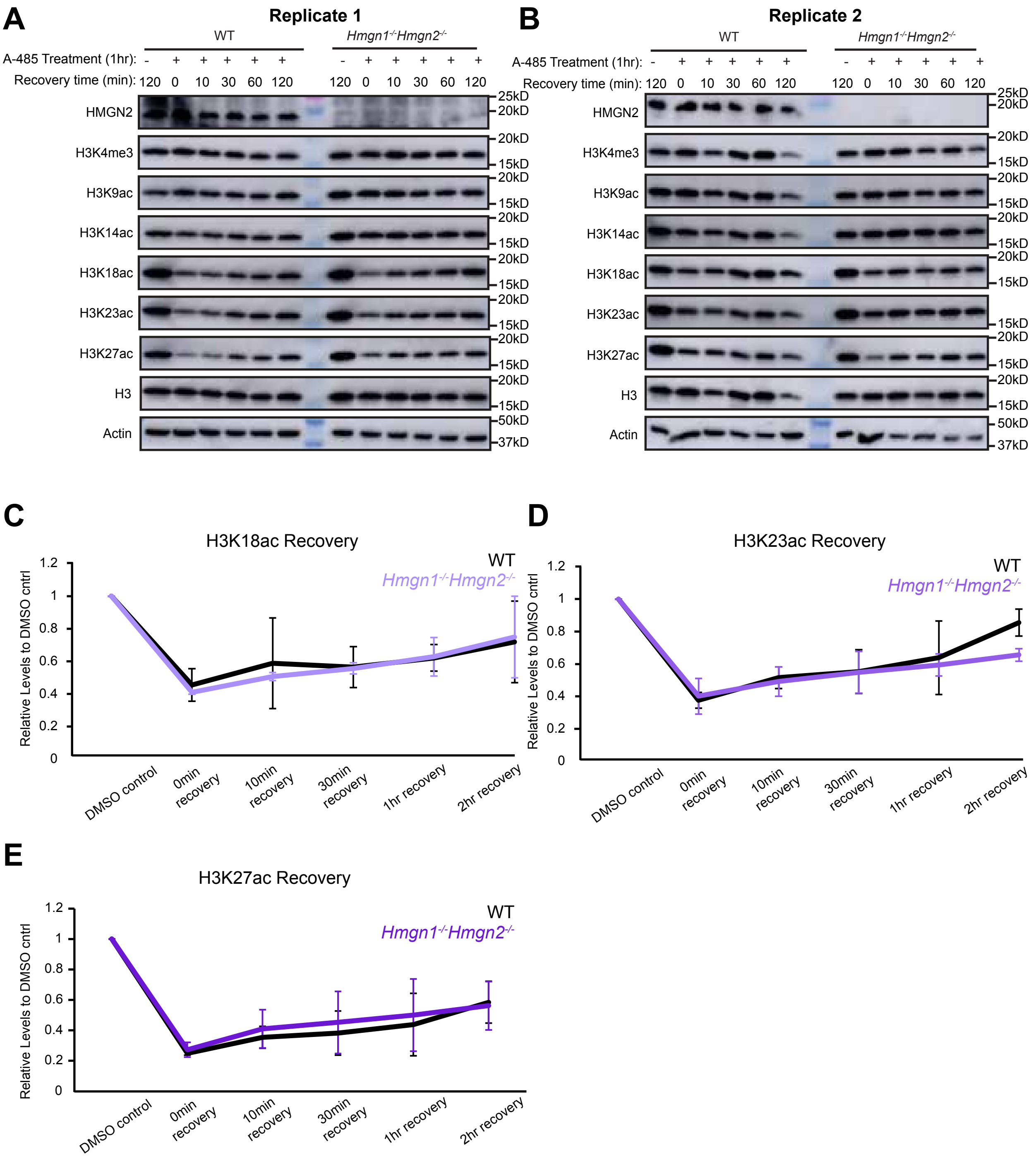

Supplementary Figure S6: HMGN1 and HMGN2 do not alter the *in vivo* recovery kinetics of p300-mediated histone acetylation.

A) Replicate 1 of Western blot analysis of H3 lysine acetylation (H3K9ac, H3K14ac, H3K18ac, H3K23ac, and H3K27ac) in *WT* mESCs and *Hmgn1*$^{-/-}$*Hmgn2*$^{-/-}$ mESCs treated with either DMSO or 10μM A-485 (p300 inhibitor) for one hour. Protein was extracted from cells collected at the 0-min recovery timepoint post-treatment or 10, 30, 60, and 120 minutes post p300 inhibitor wash out. Antibodies specific to

modified histone residues were used to quantify the levels of specific histone PTMs at each time point.

B) Replicate 2 of Western blot analysis of H3 lysine acetylation (H3K9ac, H3K14ac, H3K18ac, H3K23ac, and H3K27ac) in *WT* mESCs and *Hmgn1*$^{-/-}$*Hmgn2*$^{-/-}$ mESCs treated with either DMSO or 10µM A-485 (p300 inhibitor) for one hour. Protein was extracted from cells collected at the 0-min recovery timepoint post-treatment or 10, 30, 60, and 120 minutes post p300 inhibitor wash out. Antibodies specific to modified histone residues were used to quantify the levels of specific histone PTMs at each time point.

C) Bar graph of H3K18ac in *WT* mESCs and *Hmgn1*$^{-/-}$*Hmgn2*$^{-/-}$ mESCs in the p300 recovery experiment. Quantifications at each time point were normalized to H3 and made relative to the DMSO treated control.

D) Bar graph of H3K23ac in *WT* mESCs and *Hmgn1*$^{-/-}$*Hmgn2*$^{-/-}$ mESCs in the p300 recovery experiment. Quantifications at each time point were normalized to H3 and made relative to the DMSO treated control.

E) Bar graph of H3K27ac in *WT* mESCs and *Hmgn1*$^{-/-}$*Hmgn2*$^{-/-}$ mESCs in the p300 recovery experiment. Quantifications at each time point were normalized to H3 and made relative to the DMSO treated control.

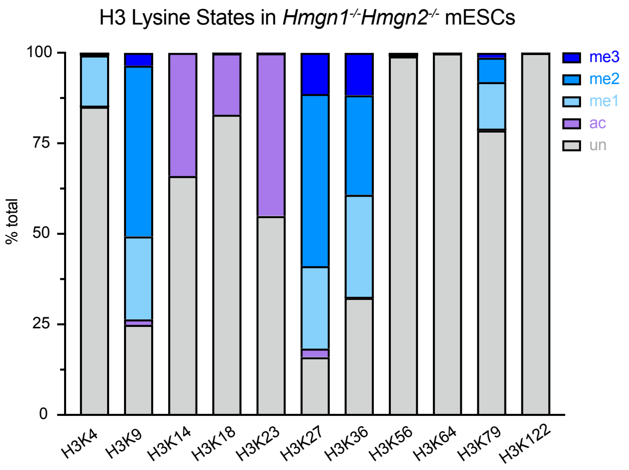

Supplementary Figure S7: Quantification of histone H3 modification states in *Hmgn1$^{-/-}$Hmgn2$^{-/-}$* mESCs by epiproteomic mass spectrometry.

Stacked bar chart showing the relative abundance of different modification states for histone H3 lysine residues in *Hmgn1$^{-/-}$Hmgn2$^{-/-}$* mESCs. Colors indicate modification types: trimethylated (dark blue), dimethylated (medium blue), monomethylated (light blue), acetylated (purple), and unmodified (gray).
